# The overlapping effects of climate change and conflict on mental health of vulnerable populations: a scoping review

**DOI:** 10.1186/s13031-026-00758-5

**Published:** 2026-02-03

**Authors:** Rawan Iriqat, Annikki Herranen-Tabibi, Zachary Sendar, Abdallah Abu-Jlambo, Wael K Al-Delaimy

**Affiliations:** 1 Herbert Wertheim School of Public Health and Human Longevity Science, University of California San Diego, 9500 Gilman Dr. MC 0628, La Jolla, 92093-0628 CA USA; 2 Department of Global Health and Social Medicine, Harvard Medical School, Boston, MA USA; 3 Department of ICU, Al-Shifa Hospital, Gaza, Palestine

**Keywords:** Climate change, Armed conflict, Mental health, Refugees, Vulnerable populations, Displacement

## Abstract

**Background:**

The mental health impacts of climate change and armed conflict are well-documented separately, yet little is known about their intersection and the compounding effects on vulnerable populations exposed to both crises.

**Aims/objective:**

This scoping review aims to map the current evidence on the combined effects of climate change and conflict-related hazards on mental health outcomes. Specifically, we categorize the pathways through which climate change and conflict interact to influence psychological well-being.

**Methods:**

We searched seven databases (PubMed, APA PsycINFO, CINAHL, Embase, Scopus, the Cochrane Library, and Google Scholar) and conducted a comprehensive gray literature search. We included populations directly affected by armed conflict and climate hazards simultaneously. Eligible studies reported mental health outcomes, including but not limited to posttraumatic stress disorder, anxiety, depression, well-being, or resilience.

**Results:**

The initial search yielded 2,865 records, 27 of which met the inclusion criteria after deduplication and screening. Populations studied aligned with the Vulnerable Populations Conceptual Model, which includes women, children, the elderly, and other high-risk groups. Slow-onset climate hazards, particularly drought, were the most frequently examined. Geographically, research was focused primarily on sub-Saharan Africa and parts of Asia. The evidence included a nearly equal distribution of conceptual and empirical studies (13 and 14 respectively), with displacement and lack of resources being the dominant pathways mediating the interaction between climate and conflict on mental health.

**Conclusion:**

Despite growing evidence, substantial gaps remain regarding the effects of climate change and conflict on mental health outside Africa and parts of Asia. Mental health initiatives should prioritize populations exposed to both climate and conflict hazards, addressing displacement, vulnerability, and resilience through integrated, context-sensitive interventions.

**Supplementary Information:**

The online version contains supplementary material available at 10.1186/s13031-026-00758-5.

## Background

Climate change and armed conflict are two of the most urgent global challenges, and their overlap significantly increases risks related to displacement, vulnerability, and health. According to the United Nations High Commissioner for Refugees (UNHCR), as of 2024, more than 120 million people are forcibly displaced worldwide, three-quarters of whom live in countries highly affected by climate change, and half are in areas facing both conflict and climate-related hazards, such as Ethiopia, Haiti, Myanmar, and Sudan [1].

Importantly, some researchers argue that climate change and armed conflict fuel and mutually reinforce each other [2, 3]. Climate hazards such as droughts, floods, and heatwaves can intensify competition over scarce resources and increase the likelihood of conflict, while conflict in turn erodes community resilience and weakens the capacity to adapt to climate stressors by destroying infrastructure, displacing populations, and weakening already fragile health systems [4, 5].

Populations affected by conflict face elevated levels of psychological distress. Research shows that prevalence rates of posttraumatic stress disorder (PTSD), depression, and anxiety are two- to three-fold higher among populations exposed to armed conflict compared to those not exposed, with women and children particularly vulnerable [6, 7]. The increasing frequency and severity of climate-related events, including droughts, floods, and heatwaves, further compound these risks, and disproportionately affect people in fragile and conflict-affected areas, contributing to elevated rates of anxiety, depression, PTSD, and ecological grief [8, 9]. Chronic climate change impacts, such as prolonged droughts and rising sea levels, also create indirect pressures that may affect mental health. These include food and water insecurity, job loss, damaged infrastructure, and forced migration [10]. Together, these conditions can weaken individual and community resilience, place additional strain on already limited health services, and increase the psychological burden on those with the fewest resources [11, 12].

Mental health is a critical yet often overlooked aspect of humanitarian response and climate adaptation efforts. Psychological well-being influences how individuals cope with displacement, rebuild after crises, and engage with support systems [13]. Unaddressed mental health conditions can worsen long-term outcomes and extend cycles of vulnerability [14]. While there is growing research on climate change and mental health, and separately on conflict and mental health, very few studies explore the combined and compounding effects of both. Additionally, there is limited understanding of how different types of climate hazards, such as sudden disasters versus slow-onset stressors, interact with conflict-related trauma to shape mental health outcomes.

This scoping review aims to map the current literature on the intersection of climate-related hazards, conflict, and mental health among vulnerable conflict-affected populations. It focuses on the types of climate hazards studied, how populations are exposed and vulnerable, the mental health outcomes reported, and the adaptation or support strategies proposed. To provide a clear structure for this complex topic, the review is guided by two complementary frameworks: the Intergovernmental Panel on Climate Change (IPCC) risk framework and the Vulnerable Populations Conceptual Model (VPCM).

### IPCC risk framework

The IPCC risk framework conceptualizes climate-related risk as the interaction of hazard, exposure, and vulnerability [15, 16]. “Hazard” refers to climate events such as droughts, floods, or heatwaves; “exposure” refers to the people or systems at risk; and “vulnerability” captures conditions that increase sensitivity and reduce coping capacity, such as poverty or displacement. Applying this framework allows the review to categorize climate hazards and examine how exposure and vulnerability contribute to mental health outcomes, particularly in populations already affected by conflict.

### Vulnerable populations conceptual model (VPCM)

The VPCM is helpful in focusing this review on populations who are not just temporarily affected but are living with ongoing challenges due to both conflict and climate stressors. This model looks at how limited resources, higher levels of risk, and poor baseline health all come together to shape vulnerability [17]. It highlights groups such as refugees, internally displaced persons, and other conflict-affected populations, emphasizing their heightened risk for long-term mental health impacts due to repeated or continuous challenges.

**Fig. 1:**
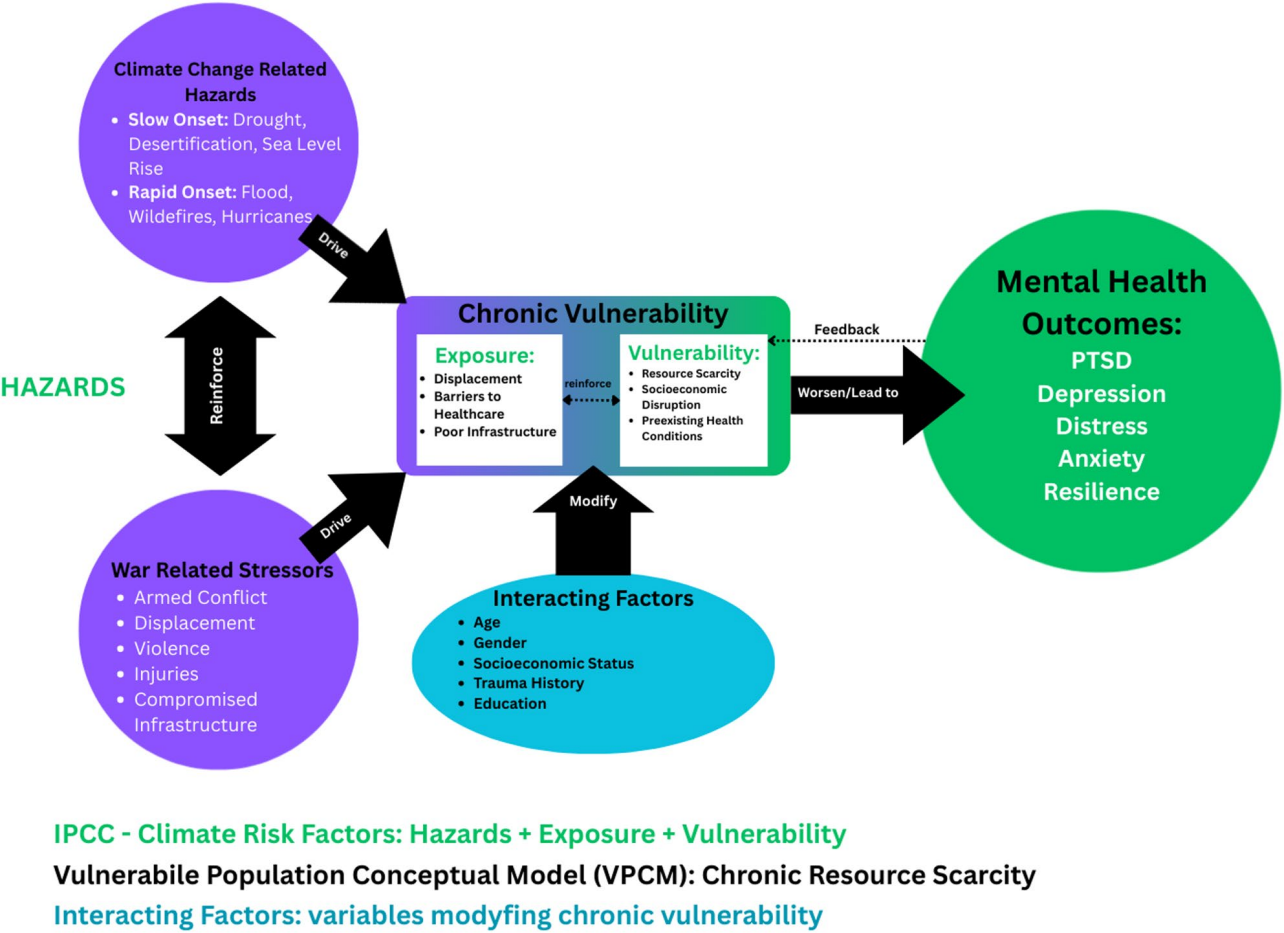
Integrated conceptual model of the IPCC risk & VPCM frameworks

Combining those frameworks allows this review to take a comprehensive approach. The IPCC framework identifies and organizes climate hazards and the populations exposed to them, while the VPCM highlights the deeper vulnerabilities and ongoing challenges faced by conflict-affected populations. Figure 1 illustrates the integrated conceptual model developed for this review, mapping the interactions between climate-related hazards, conflict stressors, and chronic vulnerability, highlighting how these pathways contribute to mental health outcomes in settings characterized by long-term resource scarcity.

## Methods

This scoping review followed established guidelines from the Preferred Reporting Items for Systematic Reviews and Meta-Analyses extension for Scoping Reviews (PRISMA-ScR) [18]. A detailed protocol was developed to guide the review process and was finalized prior to study selection and screening; however, the protocol was not registered due to the broad scope of the research question. To our knowledge, no prior protocols on this specific intersection of conflict, climate, and mental health have been registered, supporting the novelty of this work.

### Eligibility criteria

The eligibility criteria for this scoping review were guided by the Population, Concept, Context, and Outcome (PCCO) framework. Specifically, the review included studies involving populations affected by armed conflict (Population), examining the effects of climate-related hazards (Concept), within conflict-affected settings (Context), and their impact on mental health (Outcome). To maintain this focus, only studies addressing all three domains of armed conflict, climate hazards, and mental health, were included.

### Population

Eligible populations included individuals directly affected by armed conflict, including refugees, internally displaced persons (IDPs), people living in or fleeing active warzones, political violence, armed conflict, those in transit or camp settings, and communities experiencing prolonged displacement. Studies involving conflict-exposed youth, such as former child soldiers, were also included. Excluded populations included individuals living in stable settings who were not directly impacted by both conflict and climate hazards, persons displaced solely for climate or economic reasons without a history of armed conflict, and military personnel or veterans, whose experiences and support systems differ substantially from civilian populations.

### Concept: climate-related hazards

This review focuses on climate-related hazards including heatwaves, floods, droughts, dust storms, heavy precipitation, and slow-onset events such as desertification and sea-level rise. Studies explicitly tying famine, water scarcity, or food insecurity to climate-related phenomena were included. Studies focusing on non-climate environmental hazards, such as industrial pollution or geological disasters (e.g., earthquakes, tsunamis), were excluded.

### Context: conflict-affected settings

In this review, the term conflict is used broadly to encompass a range of forms of violence, including wars, civil unrest, genocide, ethnic cleansing, political violence, and local disputes, which vary in duration, scale of displacement, number of people affected, and the populations impacted [19]. Included studies examined populations living in conflict-affected contexts such as refugee or IDP camps, host communities, informal settlements, active conflict zones, or cross-border transit hubs. Research conducted in stable environments, even when climate hazards are present, was excluded unless it was directly linked to recent or ongoing conflict-related displacement.

### Outcomes: mental health

Throughout this review, “mental health outcomes” is used as an umbrella term encompassing diagnosed mental disorders, symptom-based psychological distress, and broader indicators of mental well-being, as defined in the original studies. Eligible studies therefore had to report any of the following: diagnosed or disorder-level conditions, such as PTSD, depression, anxiety disorders, complex PTSD, substance misuse, or suicidal ideation/attempts; symptom-based outcomes, including psychological distress, trauma-related symptoms, and somatic symptoms related to psychological distress; or broader psychosocial constructs, such as mental well-being or moral injury. Major psychiatric disorders (e.g., schizophrenia, bipolar disorder, or other psychotic or neurodevelopmental conditions) were excluded. Research addressing resilience, coping, or post-traumatic growth was included only if explicitly connected to mental health outcomes. Studies focusing exclusively on physical health without mental health measures were excluded.

### Study types and Language

All empirical designs were eligible, including quantitative (cross-sectional, cohort, case–control), qualitative (interviews, focus groups, ethnography), mixed methods, published ongoing study protocols, reviews, theoretical or conceptual papers, and relevant gray literature such as Non-Governmental Organization (NGO) reports and dissertations. Editorials, commentaries, and opinion pieces without empirical or conceptual contributions were excluded. Only studies published in English were considered. No restrictions were placed on publication date.

### Information sources

To capture all relevant literature, we searched seven electronic databases, PubMed, APA PsycINFO, CINAHL, Embase, Scopus, Cochrane Library, and Google Scholar, covering all records from inception to June 2025. To supplement peer-reviewed sources, we conducted targeted gray literature searches using Google’s advanced search functions to identify publicly available reports and documents from key organizations such as UNHCR, World Health Organization (WHO), and United Nations Development Programme (UNDP). Reference lists of included studies were also screened to identify additional eligible sources.

### Search strategy

An initial exploratory search was conducted to map the existing literature, to see how much research exists, what it focuses on, and where the gaps are. This step helped identify key terms, databases, and evidence gaps to guide the full scoping review. The final search strategy was later developed in collaboration with a health sciences librarian and was structured around three key domains: armed conflict, climate-related hazards, and mental health outcomes. We began by using PubMed’s advanced search builder to generate an initial list of Medical Subject Headings (MeSH) terms and free-text keywords for each domain. These terms included concepts such as “wars”, “refugees,” and “genocide” for the armed conflict domain; “climate change,” “natural disasters,” and “droughts” for the climate domain; and “mental health,” “psychological distress,” and “PTSD” for the mental health domain. Boolean operators (AND, OR) and truncation symbols (e.g., *) were used to capture relevant variations across terms. The final PubMed search strategy combined these domains using a three-part Boolean logic structure: (mental health terms) AND (climate terms) AND (armed conflict terms). Full search strings for all databases are available in Additional file 1.

### Selection of sources of evidence

All retrieved records were imported into Covidence for deduplication and screening. Title abstract screening was conducted by a primary reviewer (RI), with subsets independently reviewed by three additional reviewers (AHT, AAJ, and ZS) to ensure that each record was assessed by at least two reviewers. Conflicts were resolved through discussion or by a third reviewer (WAD). Full-text assessment of included publications was conducted independently by three reviewers (RI, ZS, AHT, and AAJ), with disagreements resolved by a third reviewer among (RI, AHT, ZS, and WAD).

### Data charting

Data extraction was performed by one reviewer (RI) and verified by two others (AHT and WAD). Data were charted using a customized form developed in Google Sheets, provided in Additional file 2. The charting form was pilot-tested on a subset of studies to ensure appropriateness of the planned data synthesis. Extracted data items are outlined in Table 1.

**Table 1:** Data items to be extracted

Data Item	Description
**Record ID**	Unique identifier assigned to each record
**Citation**	Reference details of the study, including first author’s name and year of publication.
**Region/Country**	Geographic focus of the study/record.
**Population**	Description of the population involved (e.g., refugees, IDPs, children, host communities).
**Climate Hazard**	Specific climate-related hazards discussed with attention to slow-onset vs. rapid onset
**Armed Conflict Context**	Description of the armed conflict or displacement context (e.g., active warzone, post-conflict setting).
**Mental Health Focus**	Mental health outcomes assessed/reported (e.g., PTSD, depression, anxiety, distress, resilience).
**Mechanisms/Pathways**	Described pathways linking conflict and climate hazards to mental health outcomes (e.g., displacement stressors, livelihood loss, food insecurity due to drought, etc.)
**Interventions & Mitigation Strategies Proposed (if any)**	Any proposed or implemented strategies to address mental health impacts in the studied population.
**Key insights related to our research question**	Summary of the study’s main findings or contributions relevant to the intersection of interest, and how the study aligns with/contributes to the main review question.

### Data synthesis

The results were synthesized narratively and structured around the five guiding sub-questions of the review. Specifically, the synthesis examine: the geographic regions and population groups represented in the literature; the type and frequencies of climate-related hazards studied; the mental health outcomes reported; the characteristics of conflict exposure and how these intersect with climate-related stressors; and the pathways, needs, or responses described, including any adaptation or mitigation strategies.

The data extracted from the included studies were first grouped and summarized descriptively to identify patterns related to study design or type of record to distinguish between conceptual and empirical contributions. The geographic regions covered in the included studies, types of climate hazards, and associated mental health outcomes were also mapped to highlight concentrations of evidence and gaps.

Following this descriptive phase, conceptual frameworks were applied to deepen the understanding of the interactions between climate change and conflict. The IPCC risk framework was used to conceptualize population risk as a function of hazard, exposure, and vulnerability. The VPCM was used to identify vulnerable populations, and pathways through which climate and conflict jointly affect their mental health. Drawing on these two frameworks, we structured the findings around two main domains: pathways of interaction that mediate the vulnerability of the affected population and their needs. This integration of frameworks helps highlight how population characteristics, climate hazards, and conflict exposure interact to heighten vulnerability to mental health challenges and clarifies the resulting needs that arise from this vulnerability.

## Results

### Selection of sources of evidence

**Fig. 2:**
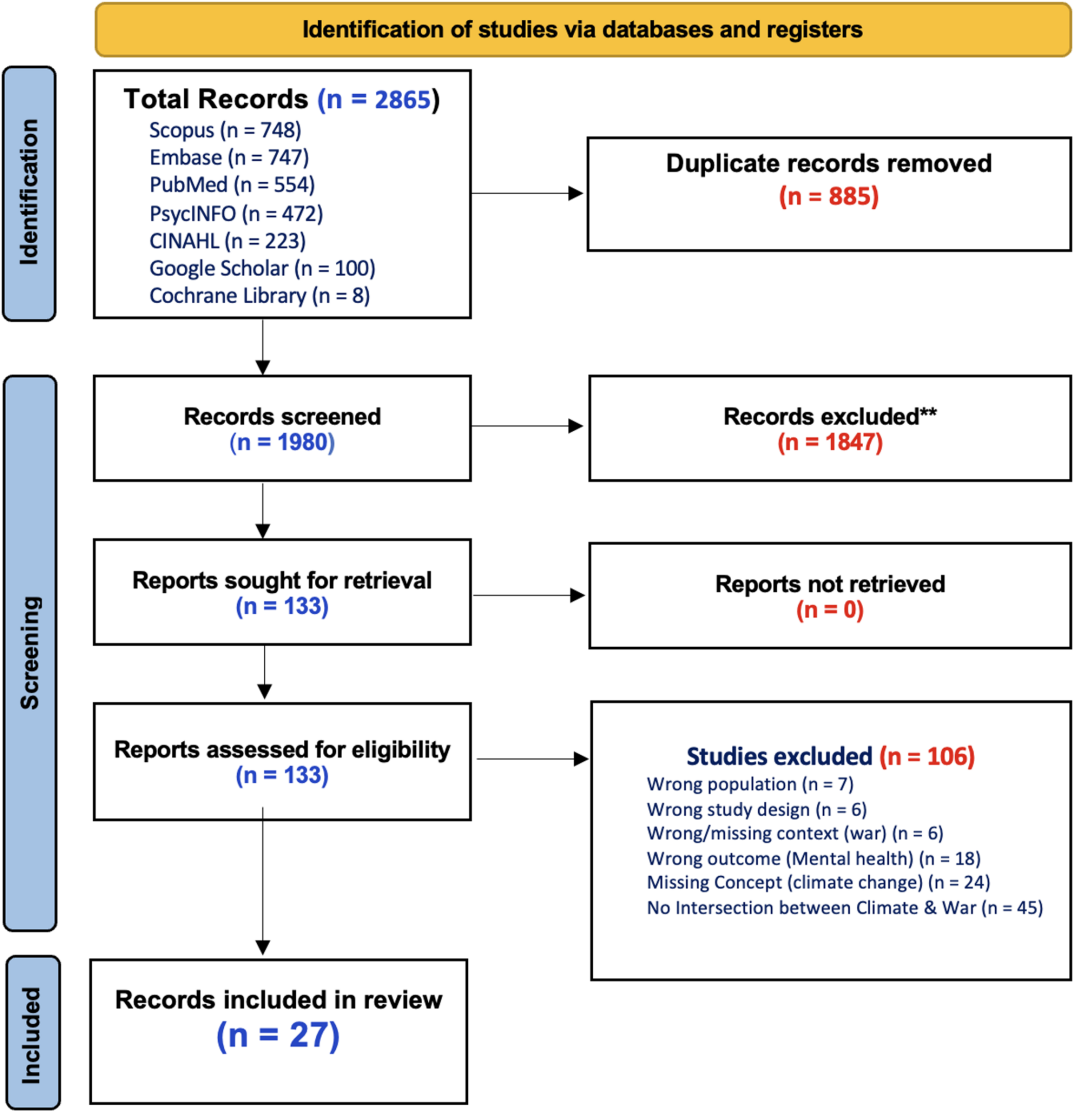
PRISMA flowchart

A total of 2,865 records were imported into Covidence for screening. After removing 885 duplicates, 1,980 records remained for title and abstract screening. Of these, 1,847 were excluded as irrelevant, and 133 articles were retained for full-text screening. Following full-text review, 106 studies were excluded for reasons such as lack of climate–conflict intersection, wrong population, missing conflict context or climate concept. Figure 2 shows the PRISMA flow chart for the screening process [20].

### Study characteristics

#### Record type/design

The included records employed a variety of methodological approaches. Fourteen studies provided empirical evidence [21–34], including qualitative interviews, cross-sectional surveys, mixed-methods research, and case studies. The remaining 13 studies were conceptual [35–47], including conceptual frameworks, perspectives, literature reviews, narrative reviews, book chapters, and commentaries. This distribution reflects a combination of direct evidence from affected populations and broader conceptual analyses of the intersection.

#### Geographic distribution of included records

**Fig. 3:**
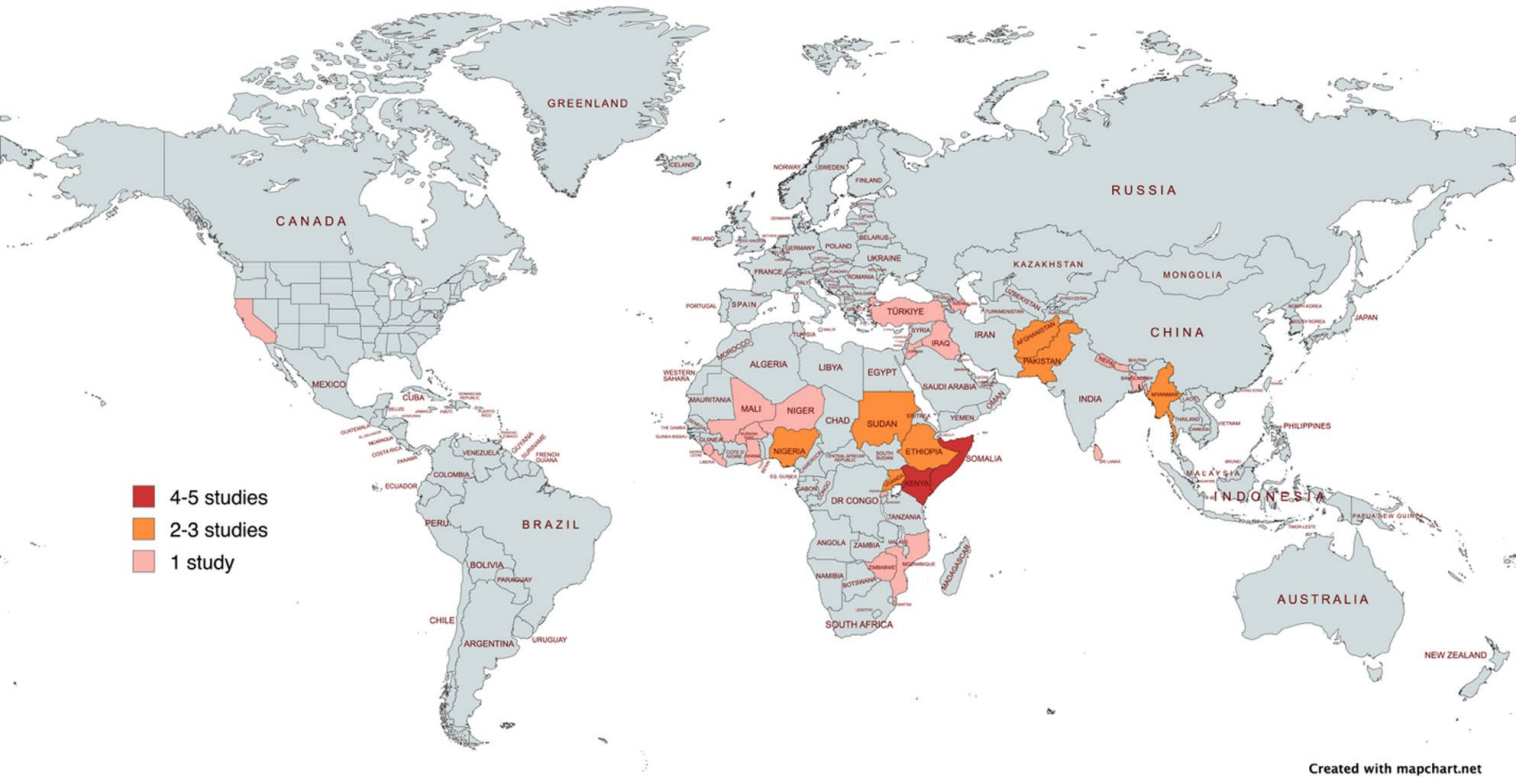
Geographic distribution of countries covered in the studies

The literature presented in this review spans multiple regions, with the majority (*n* = 16) report on Africa [21–25, 27–30, 33, 35, 38, 41, 43–45], followed by Asia [26, 31, 32, 34, 36, 37, 40, 42, 47]. Some studies included multiple countries or broader regional perspectives. As shown in Fig. 3, evidence is concentrated in sub-Saharan Africa, whereas other regions remain underrepresented.

#### Affected populations

The populations in the included records consistently highlight groups disproportionately affected by conflict, displacement, and climate-related hazards. These include refugees, IDPs, and immigrants [21–24, 27, 29, 31–38, 41–43, 47]. With particular emphasis on individuals facing compounded vulnerabilities, such as pregnant and perinatal women, caregivers of orphans, mothers of malnourished children, and pastoralist adolescents, and children [21, 24, 25, 28, 30, 32, 33, 35–37, 46, 47].

#### Types of climate hazards and their associated mental health outcomes

Across the included studies, climate hazards ranged from rapid-onset events (e.g., cyclones, floods, heatwaves, landslides) [22, 24, 26, 27, 29, 31, 34, 36, 40, 42, 43, 46, 47] to slow-onset phenomena (e.g., drought, desertification, rising temperatures, food and water insecurity) [21, 33, 35, 38, 39, 41, 46], all of which represent the hazard domain of the IPCC risk framework. These hazards were often compounded by conflict or war-related contexts, including civil wars, ethnic violence, political oppression, prolonged military operations, and forced displacement, which indicate exposure to dire contexts as per the IPCC risk framework. Slow-onset hazards are more frequently studied than rapid-onset events, with both types reported in the literature as being associated with adverse mental health outcomes, including depression, anxiety, PTSD, psychological distress, and reduced well-being. Rapid-onset events have been largely documented in Asia, such as Myanmar and Afghanistan, whereas slow-onset events have been highlighted in the African continent, such as Somalia, Sudan and Ethiopia. Additional file 4: Table 2 outlines the main characteristics of each included record along with extracted key data items.

#### Pathways of interaction (mediators of vulnerability)

The primary pathways linking conflict and climate hazards to adverse mental health outcomes centered on a few key mediators. Forced displacement, mentioned in 23 of the 27 studies [21, 22, 24, 26, 35, 37, 38, 40–47], and poor living conditions, including overcrowding and inadequate shelter, were frequently described as contributing to worse mental health outcomes. Resource scarcity, particularly food and water insecurity, loss of livelihood, and economic instability, were described almost evenly across empirical and conceptual papers as factors intensifying stress and limiting coping capacity [21, 23, 25–28, 30–33, 35, 38–45, 47]. Limited access to healthcare, social support, and other essential services were also reported to heighten vulnerability, especially among women, adolescents, children, and caregivers, and the evidence mostly comes from empirical studies [25, 28, 31–33, 35, 41]. Additionally, exposure to trauma and abuse, such as sexual and gender-based violence and inequities, emerged as a compounding factor worsening mental health outcomes [21, 25, 26, 28–30, 33–37, 39, 40, 42, 43, 45]. Additional file 5: Table 3 summarizes the pathways in which climate hazards and armed conflicts are shown to affect mental health outcomes of vulnerable populations in the included records, as well as key findings related to the research questions of the review.

#### Population needs

Several key needs emerge for populations affected by the intersection of armed conflict and climate-related hazards. First, there is a strong emphasis in the literature on basic needs and resource support, such as food security, water access, shelter, and climate-resilient livelihood programs, which are often framed as essential for mitigating mental health consequences, and are mostly drawn from empirical studies [21, 25, 28, 31, 33, 35, 38, 44]. Second, pre-established mental health and psychosocial support (MHPSS) is consistently highlighted, including community-based and culturally adapted programs, group therapy, tele-psychiatry, and education for local healthcare providers to reduce stigma and improve service delivery, stemming from both empirical and conceptual studies alike [24, 26, 29, 30, 33, 35–37, 39–41]. Third, policy and structural interventions, including national mental health strategies, long-term disaster preparedness, climate governance, gender-based violence prevention, and the integration of mental health into primary care, are frequently recommended in conceptual papers, and in a few empirical studies [27, 31, 33, 35, 36, 38, 39, 43, 44, 46, 47].

## Discussion

This study aimed to map the current evidence on the intersection of climate change and armed conflict, and their impacts on mental health outcomes of vulnerable populations affected by the overlapping crises. Despite a broad and systematic search across multiple databases, only 27 studies explicitly addressed the mental vulnerability of populations affected by both armed conflict and climate change (14 empirical and 13 conceptual). Additionally, the geographic distribution was uneven, with research concentrated in sub-Saharan Africa and Southeast Asia, while regions such as Gaza and Ukraine, which face overlapping armed conflict and climate risks, remain underrepresented [48, 49]. A gap likely due to barriers such as restricted access, safety and ethical concerns, and disrupted research and health infrastructures. The lack of evidence from these settings should not be interpreted as a lack of need or impact; rather, it highlights limitations in the current evidence base. These gaps and resulting knowledge imbalances indicate that future research should prioritize empirical studies in underrepresented regions and contexts, particularly in both active conflict and post-war settings. Such studies should focus on both acute and long-term mental health outcomes. Strengthening the evidence base will also require the use of integrated exposure measures, longitudinal follow-up, and mixed-methods approaches that capture cumulative and lived experiences.

### VPCM and IPCC risk framework domains

Beyond mapping the existing evidence, this review advances conceptual understanding of the intersection between climate change and armed conflict. Findings were mapped to IPCC risk framework and VPCM domains, allowing us to identify interactions between environmental hazards, exposure, vulnerability, and structural constraints that drive mental health outcomes. This integration of frameworks highlights mental health vulnerability as a dynamic process shaped by environmental hazards, sociopolitical instability, and long-term structural constraints, rather than as the result of isolated exposures.

Across the included studies, the focus on refugees, internally displaced persons (IDPs), and other conflict-affected populations, particularly women, children, adolescents, and older adults, including those facing compounded vulnerabilities such as pregnant and perinatal women, caregivers of orphans, and mothers of malnourished children, aligns with previous research highlighting these groups as especially vulnerable to the combined effects of conflict and climate hazards [18, 50, 51]. The focus on slow-onset hazards, particularly drought and its effects on food and water security, reflects their chronic and pervasive impact on mental health, consistent with previous research [52, 53]. Similarly, the attention to floods among rapid-onset hazards aligns with prior studies identifying PTSD, depression, and anxiety as key mental health outcomes following flood exposure in conflict-affected and displaced populations [54]. Armed conflict contexts span decades-long civil wars, political instability, ethnic and religious violence, and state-sponsored oppression, often resulting in displacement and prolonged exposure to stressful living conditions. These findings illustrate the interplay of hazard, exposure, and vulnerability as defined by the IPCC risk framework, while also highlighting the chronic adversity and resource limitations emphasized in the VPCM, demonstrating how predisposing factors and prolonged stressors shape mental health outcomes in conflict-affected populations. Conceptually, this integration solidifies that climate-related mental health risks in conflict-affected settings cannot be fully understood through hazard exposure alone. Instead, mental health outcomes emerge from the interaction between environmental stressors (IPCC) and entrenched social, economic, and political vulnerabilities (VPCM). This framing clarifies why similar climate hazards may produce disproportionately severe mental health impacts in conflict-affected populations compared to more stable contexts.

### Enabling pathways

The integration of frameworks in this review highlights feedback loops in which climate hazards and conflict perpetuate mental health risk, particularly through repeated displacement, prolonged insecurity, and erosion of protective social structures. Displacement emerged as a central pathway linking the effects of climate hazards and conflict to adverse mental health outcomes. This aligns with prior evidence showing that populations forced to relocate due to war, floods, droughts, or other hazards face disruptions to social networks, limited access to health care, and overcrowded living conditions, with social stigma further compounding mental health risks [4, 55, 56]. Resource scarcity, particularly food and water insecurity, loss of livelihood, and economic instability, may amplify stress and reduces coping abilities, reflecting the chronic impacts of slow-onset climate hazards like drought and desertification [21]. Some studies highlight the heightened risk of trauma and abuse, including sexual and gender-based violence, affecting minors and women in contexts of conflict and climate-related hazards [30, 37]. This compounded vulnerability is further supported by a systematic review documenting the specific risks faced by women and young girls in humanitarian settings [57]. Similarly, other research shows that conflict and climate-related crises worsen gender inequalities and increase the risk of sexual and gender-based violence for women and children, as chaos, displacement, and social disruption increase their vulnerability to trauma and abuse [58]. Together, these pathways, again, demonstrate that mental health outcomes are rarely the result of single exposures but are shaped by the overlapping effects of climate hazards and conflict. These findings suggest that mental health interventions in climate–conflict settings must be pathway-specific and context-sensitive, recognizing that different climate hazards and conflict dynamics generate distinct mechanisms of psychological harm. As a result, standardized interventions may fail to address key sources of distress and could exacerbate inequities. Future programs should therefore target these enabling pathways directly, for example, through interventions addressing food and water insecurity, access to safe shelter, and addressing the issue of gender-based violence in displacement settings.

### Relevance to future impacts and implications

The growing frequency and intensity of armed conflicts through long term protracted and destructive wars, as now witnessed in Gaza and Ukraine [48, 49], where civilian infrastructure is intentionally targeted as retaliation or punishment to the general population [59–62], have heightened the vulnerability of global populations. This vulnerability may be further exacerbated by climate change, which disproportionately affects the health of already at-risk populations. Even in the absence of armed conflict, climate-related psychological impacts are projected to be among the most significant and immediate adverse health outcomes [4]. When combined together, we are facing a compounded public mental health crisis. And the current situation in Sudan exemplifies this overlap. The ongoing civil war has displaced more than 11 million people and left nearly two-thirds of the population in need of humanitarian assistance [63]. Recent floods in 2025 linked to heavy rains and water management failures displaced thousands more in already war-torn areas [64], illustrating how climate hazards deepen vulnerability, create secondary displacement, and have the potential to heighten risks of poor mental health outcomes. These mental health effects often persist beyond the immediate crisis, affecting long-term outcomes for individuals, families, and broader societal functioning, including economic stability, education, and community cohesion.

Much of the literature included in this review suggests that the combined effects of climate change and conflict are most often reported in low- and middle-income countries. These settings are frequently described as facing greater vulnerability and more limited capacity to respond to overlapping crises. The findings also suggest that conflict-affected populations in climate-vulnerable regions may experience a higher mental health burden, pointing to the importance of equity-centered approaches to climate and mental health governance. These mental health impacts are commonly discussed within broader contexts of structural and geopolitical inequities, indicating that responses may need to extend beyond the health sector alone.

Looking ahead, mental health programming and research must shift toward polycrisis-informed frameworks that account for the cumulative, interacting effects of armed conflict and climate-related hazards over time, rather than viewing recovery as distinct, separate stages. As a first step, mental health assessment tools in global humanitarian settings could benefit from integrating supplementary components that capture climate-related stressors and vulnerabilities, ensuring mental health outcomes are understood not only as consequences of climate–conflict exposure, but also as indicators of compounded vulnerability. Additionally, integrating mental health monitoring into climate adaptation, disaster preparedness, and humanitarian early warning systems could improve preventive strategies and reduce subsequent harm. Moreover, the evidence that mental health may be exacerbated by lack of basic needs also suggests that mental health support must be structurally embedded within climate adaptation, humanitarian assistance, and displacement governance, ensuring that psychosocial well-being is treated as a core outcome of multisectoral interventions at both community and individual levels.

### Limitations

This review has several limitations. First, despite a comprehensive search strategy, relevant studies may have been missed, particularly those published in languages other than English or in journals not indexed in the databases we searched. Second, data extraction was conducted by a single reviewer and verified by two additional reviewers. Although this process helps ensure consistency, having multiple reviewers independently extract data could have further minimized the risk of bias. Finally, the use of the VPCM framework early in the review meant that our selection focused on populations traditionally considered vulnerable, which limited inclusion of groups that do not neatly fall within the framework. For example, populations affected by war-like acts in more stable, high-income contexts, such as 9/11 survivors exposed to subsequent hurricanes, experience both conflict-related trauma and climate hazards but may not display the chronic resource limitations or vulnerabilities emphasized in the VPCM. We also excluded several veterans and military studies which may nearly fit the framework in terms of exposure and potential psychological consequences, but they differ in relative risk and resource availability. Those populations typically have access to healthcare and support systems, meaning they do not meet the VPCM domain of limited resource availability that compounds vulnerability. As a result, such populations were excluded, and certain insights regarding the mental health impacts of combined climate and conflict exposures in well-supported populations may have been overlooked.

## Conclusion

By articulating the pathways through which climate hazards and conflict interact to shape mental health outcomes, this review offers a conceptual foundation to guide future empirical research, intervention design, and climate-informed mental health policy. To the best of our knowledge, this is the first scoping review to examine how the overlap of climate change and armed conflict exacerbates vulnerability of the population to the psychological impacts of these large-scale risks. Evidence suggests that climate change may impact health in general, and mental health more specifically, and could contribute to health disparities by disproportionately affecting the most vulnerable in society. Countries most at risk of these disparities are often those experiencing the impacts of armed conflicts and climate-related adversities. Prevention strategies should be prioritized by international communities to develop climate and mental health adaptation interventions and policies prior to these extreme climate events or conflicts. Expanding evaluations of these measures in real-world settings is urgently needed to better support those most affected in the Global South and to generate insights that are equally critical for the Global North, as illustrated by the ongoing situation in Ukraine.

## Supplementary Information

Additional file 1: Search strategy

Additional file 2: Data Extraction Form

Additional file 3: PRISMA-ScR-Checklist

Additional file 4: Table 2- Study Characterstics of Included Articles

Additional file 5: Table 3- Pathways of Interaction

## Data Availability

All data generated or analyzed during this study are included in this published article and its additional files.
