# Supplementary material for: The overlapping effects of climate change and conflict on mental health of vulnerable populations: a scoping review
Source: Confl Health. 2026 Feb 3;20:21. doi: 10.1186/s13031-026-00758-5 (PMC12955018; doi:10.1186/s13031-026-00758-5)
Supplement: Supplementary file 1 — Additional file 1: Search strategy [file 13031_2026_758_MOESM1_ESM.pdf]

Additional file 1 – Search Strategy

Search strategy by database

Pubmed Search Terms:

WAR:
"Armed Conflicts"[MeSH Terms]
OR "refugee camp*"[MeSH Terms]
OR "Warfare and Armed Conflicts"[MeSH Terms]
OR "armed conflict*"[tiab]
OR "War"[tiab]
OR "wars"[tiab]
OR "Refugees"[MeSH Terms]
OR "Internally Displaced Persons"[tiab]
OR "displaced people"[tiab]
OR "refugee*"[tiab]
OR "Genocide"[MeSH Terms]
OR "Terrorism"[MeSH Terms]
OR "Genocide"[tiab]
OR "Terrorism"[tiab]

CLIMATE:
"Natural Disasters"[MeSH Terms]
OR "Climate Change"[MeSH:noexp]
OR "Global Warming"[MeSH Terms]
OR "Climatic Processes"[MeSH Terms]
OR "Sea Level Rise"[MeSH Terms]
OR "Water Insecurity"[MeSH Terms]
OR "Extreme Heat"[MeSH Terms]
OR "Droughts"[MeSH Terms]
OR "Floods"[MeSH Terms]
OR "Desertification"[tiab]
OR "soil degradation"[tiab]
OR "Food Supply"[MeSH Terms]
OR "Climate Change"[tiab]
OR "Global Warming"[tiab]
OR "natural disaster*"[tiab]
OR "drought*"[tiab]
OR "flood*"[tiab]
OR "heat wave*"[tiab]
OR "water scarcity"[tiab]
OR "water shortage*"[tiab]
OR "environmental stress"[tiab]

MENTAL HEALTH:
("Mental Health"[MeSH Terms]

OR "Anxiety Disorders"[MeSH Terms]
OR "Anxiety"[MeSH:noexp]
OR "Catastrophization"[MeSH Terms]
OR "Anxiety"[tiab]
OR "Mental Health"[tiab]
OR "Psychological Distress"[MeSH Terms]
OR "catastrophiz*"[tiab]
OR "resilience, psychological"[MeSH Terms]
OR "stress disorders, post traumatic"[MeSH Terms]
OR "Depressive Disorder"[MeSH Terms]
OR "PTSD"[tiab]
OR "depression"[tiab]
OR "Psychological Distress"[tiab]
OR "resilien*"[tiab]
OR "coping"[tiab]
OR "psychological trauma"[tiab]

Final PUBMED Search:

(("Mental Health"[MeSH Terms] OR "Anxiety Disorders"[MeSH Terms] OR "Anxiety"[MeSH:noexp] OR "Catastrophization"[MeSH Terms] OR "Anxiety"[tiab] OR "Mental Health"[tiab] OR "Psychological Distress"[MeSH Terms] OR "catastrophiz*"[tiab] OR "resilience, psychological"[MeSH Terms] OR "stress disorders, post traumatic"[MeSH Terms] OR "Depressive Disorder"[MeSH Terms] OR "PTSD"[tiab] OR "depression"[tiab] OR "Psychological Distress"[tiab] OR "resilien*"[tiab] OR "coping"[tiab] OR "psychological trauma"[tiab]) AND ("Natural Disasters"[MeSH Terms] OR "Climate Change"[MeSH:noexp] OR "Global Warming"[MeSH Terms] OR "Climatic Processes"[MeSH Terms] OR "Sea Level Rise"[MeSH Terms] OR "Water Insecurity"[MeSH Terms] OR "Extreme Heat"[MeSH Terms] OR "Droughts"[MeSH Terms] OR "Floods"[MeSH Terms] OR "Desertification"[tiab] OR "soil degradation"[tiab] OR "Food Supply"[MeSH Terms] OR "Climate Change"[tiab] OR "Global Warming"[tiab] OR "natural disaster*"[tiab] OR "drought*"[tiab] OR "flood*"[tiab] OR "heat wave*"[tiab] OR "water scarcity"[tiab] OR "water shortage*"[tiab] OR "environmental stress"[tiab])) AND ("Armed Conflicts"[MeSH Terms] OR "refugee camp*"[MeSH Terms] OR "Warfare and Armed Conflicts"[MeSH Terms] OR "armed conflict*"[tiab] OR "War"[tiab] OR "wars"[tiab] OR "Refugees"[MeSH Terms] OR "Internally Displaced Persons"[tiab] OR "displaced people"[tiab] OR "refugee*"[tiab] OR "Genocide"[MeSH Terms] OR "Terrorism"[MeSH Terms] OR "Genocide"[tiab] OR "Terrorism"[tiab])

Psycinfo Search Terms:

Additional file 1 – Search Strategy

WAR:

(DE "War" OR DE "Refugees" OR DE "Genocide" OR DE "Terrorism" OR DE "Political Revolution") OR (XB "War" OR XB "Refugees" OR XB "Armed Conflict" OR XB "Internally Displaced Persons" OR XB "Genocide" OR XB "Displaced Persons" OR XB "Terrorism" OR XB "Refugee Camps")

CLIMATE:

(DE "Natural Disasters" OR DE "Global Warming" OR DE "Climate Anxiety" OR DE "Extreme Weather" OR DE "Heat Effects" OR DE "Food Insecurity" OR DE "Environmental Stress" OR DE "Environmental Effects" OR DE "Seasonal Variations" OR DE "Temperature Effects") OR (XB "Natural Disasters" OR XB "Global Warming" OR XB "Environmental Stress" OR XB "Climate Anxiety")

MENTAL HEALTH:

(DE "Mental Disorders" OR DE "Mental Health" OR DE "Psychological Stress" OR DE "Resilience (Psychological)" OR DE "Anxiety" OR DE "Depression" OR DE "Posttraumatic Stress Disorder" OR DE "Suicide Risk Assessment" OR DE "Psychosocial Outcomes" OR DE "Mental Health Screening" OR DE "Psychological Distress") OR (XB "Mental Health" OR XB "Psychological Well-Being" OR XB "Psychological Resilience" OR XB "Suicide Risk" OR XB "Depression" OR XB "Anxiety")

Final PSYCINFO Search:

(((DE "War" OR DE "Refugees" OR DE "Genocide" OR DE "Terrorism" OR DE "Political Revolution") OR (XB "War" OR XB "Refugees" OR XB "Armed Conflict" OR XB "Internally Displaced Persons" OR XB "Genocide" OR XB "Displaced Persons" OR XB "Terrorism" OR XB "Refugee Camps")) AND ((DE "Natural Disasters" OR DE "Global Warming" OR DE "Climate Anxiety" OR DE "Extreme Weather" OR DE "Heat Effects" OR DE "Food Insecurity" OR DE "Environmental Stress" OR DE "Environmental Effects" OR DE "Seasonal Variations" OR DE "Temperature Effects") OR (XB "Natural Disasters" OR XB "Global Warming" OR XB "Environmental Stress" OR XB "Climate Anxiety")) AND ((DE "Mental Disorders" OR DE "Mental Health" OR DE "Psychological Stress" OR DE "Resilience (Psychological)" OR DE "Anxiety" OR DE "Depression" OR DE "Posttraumatic Stress Disorder" OR DE "Suicide Risk Assessment" OR DE "Psychosocial Outcomes" OR DE "Mental Health Screening" OR DE "Psychological Distress") OR (XB "Mental Health" OR XB "Psychological Well-Being" OR XB "Psychological Resilience" OR XB "Suicide Risk" OR XB "Depression" OR XB "Anxiety"))

CINAHL Search Terms:

Additional file 1 – Search Strategy

WAR:

MH "War+" OR MH "Refugees" OR MH "Terrorism+" OR (TI war OR AB war OR KW war OR TI refugee* OR AB refugee* OR KW refugee* OR TI terrorism OR AB terrorism)

CLIMATE:

(MH "Natural Disasters+" OR MH "Climate Change+" OR MH "Environmental Justice" OR MH "Climate Anxiety" OR (TI climate* OR AB climate* OR KW climate* OR TI "global warming" OR AB "global warming")

MENTAL HEALTH

MH "Mental Disorders+" OR MH "Psychological Trauma" OR MH "Stress Disorders" OR MH "Depression" OR MH "Anxiety Disorders" OR (TI mental* OR AB mental* OR KW mental* OR TI psych* OR AB psych* OR KW psych*)

Final CINAHL Search:

(MH "Mental Disorders+" OR MH "Psychological Trauma" OR MH "Stress Disorders" OR MH "Depression" OR MH "Anxiety Disorders" OR (TI mental* OR AB mental* OR KW mental* OR TI psych* OR AB psych* OR KW psych*) AND (MH "Natural Disasters+" OR MH "Climate Change+" OR MH "Environmental Justice" OR MH "Climate Anxiety" OR (TI climate* OR AB climate* OR KW climate* OR TI "global warming" OR AB "global warming") AND (MH "War+" OR MH "Refugees" OR MH "Terrorism+" OR (TI war OR AB war OR KW war OR TI refugee* OR AB refugee* OR KW refugee* OR TI terrorism OR AB terrorism)

SCOPUS Search Terms

Mental Health:

TITLE-ABS-KEY: "mental health" OR "anxiety" OR "depression" OR "catastrophization" OR "psychological distress" OR "resilience" OR "post-traumatic stress" OR "PTSD" OR "coping" OR "psychological trauma" OR "suicide" OR "psychosocial outcomes" OR "psychological well-being"

WAR:

TITLE-ABS-KEY "armed conflict*" OR "war" OR "wars" OR "warfare" OR "refugees" OR "refugee*" OR "refugee camps" OR "internally displaced persons" OR "displaced people" OR "genocide" OR "terrorism"

CLIMATE:

Additional file 1 – Search Strategy

TITLE-ABS-KEY: "natural disasters" OR "climate change" OR "global warming" OR "climatic processes" OR "sea level rise" OR "water insecurity" OR "extreme heat" OR "drought*" OR "flood*" OR "desertification" OR "soil degradation" OR "food supply" OR "heat wave*" OR "water scarcity" OR "water shortage*" OR "environmental stress" OR "environmental effects" OR "seasonal variations" OR "temperature effects"

KW: War OR "ARMED CONFLICT" OR "CLIMATE" OR "Global warming"

Final SCOPUS Search:

TITLE-ABS-KEY ("mental health" OR "anxiety" OR "depression" OR "PTSD" OR "catastrophiz*" OR "mental health" OR "psychological distress" OR "resilience" OR "post-traumatic stress" OR "depression" OR "PTSD" OR "coping" OR "psychological trauma" OR "suicide" OR "psychosocial outcomes" OR "mental health screening" OR "psychological well-being") AND TITLE-ABS-KEY ("natural disasters" OR "climate change" OR "global warming" OR "climatic processes" OR "sea level rise" OR "water insecurity" OR "extreme heat" OR "drought*" OR "flood*" OR "desertification" OR "soil degradation" OR "food supply" OR "heat wave*" OR "water scarcity" OR "water shortage*" OR "environmental stress" OR "environmental effects" OR "seasonal variations" OR "temperature effects") AND TITLE-ABS-KEY ("armed conflict*" OR "war" OR "wars" OR "warfare" OR "refugees" OR "refugee*" OR "refugee camps" OR "internally displaced persons" OR "displaced people" OR "genocide" OR "terrorism" OR "political revolution") AND TITLE-ABS-KEY ("refugees" OR "displaced persons")

CHOCHRANE

Mental Health

(MeSH descriptor: [Mental Health] explode all trees OR MeSH descriptor: [Anxiety Disorders] explode all trees OR (mental health):ti,ab,kw OR MeSH descriptor: [Anxiety] explode all trees OR MeSH descriptor: [Catastrophization] this term only OR MeSH descriptor: [Climate Anxiety] explode all trees OR MeSH descriptor: [Depressive Disorder] explode all trees OR (Anxiety OR catastrophiz* OR Resilien* OR PTSD OR depression OR coping):ti,ab,kw)

AND

War

Additional file 1 – Search Strategy

(MeSH descriptor: [Warfare and Armed Conflicts] explode all trees OR MeSH descriptor: [Refugees] this term only OR MeSH descriptor: [Genocide] explode all trees OR (Internally Displaced Persons):ti,ab,kw OR (WAR OR WARS):ti,ab,kw OR (Refugees OR displaced people OR refugee* OR Genocide OR Terrorism):ti,ab,kw)

AND

Climate Change

(MeSH descriptor: [Natural Disasters] explode all trees OR MeSH descriptor: [Climate Change] explode all trees OR MeSH descriptor: [Global Warming] explode all trees OR (natural disaster*):ti,ab,kw OR (climate change):ti,ab,kw OR (global warming):ti,ab,kw OR (Desertification OR "soil degradation" OR drought* OR flood*):ti,ab,kw)

Final cochrane search

("Mental Health"[MeSH] OR "Anxiety Disorders"[MeSH] OR "mental health":ti,ab,kw OR "Anxiety"[MeSH] OR "Catastrophization"[MeSH] OR "Climate Anxiety"[MeSH] OR "Depressive Disorder"[MeSH] OR Anxiety:ti,ab,kw OR catastrophiz:ti,ab,kw OR Resilien:ti,ab,kw OR PTSD:ti,ab,kw OR depression:ti,ab,kw OR coping:ti,ab,kw) AND ("Warfare and Armed Conflicts"[MeSH] OR "Refugees"[MeSH] OR "Genocide"[MeSH] OR "Internally Displaced Persons":ti,ab,kw OR WAR:ti,ab,kw OR WARS:ti,ab,kw OR Refugees:ti,ab,kw OR displaced people:ti,ab,kw OR refugee*:ti,ab,kw OR Genocide:ti,ab,kw OR Terrorism:ti,ab,kw) AND ("Natural Disasters"[MeSH] OR "Climate Change"[MeSH] OR "Global Warming"[MeSH] OR natural disaster*:ti,ab,kw OR climate change:ti,ab,kw OR global warming:ti,ab,kw OR Desertification:ti,ab,kw OR "soil degradation":ti,ab,kw OR drought*:ti,ab,kw OR flood*:ti,ab,kw)**

Google scholar (first 100 results)

wars OR "armed conflict" OR genocide OR terrorism "climate change" OR "global warming" OR "natural disaster" "mental health" OR anxiety OR PTSD OR depression

Embase

WAR:

Additional file 1 – Search Strategy

'war'/exp OR 'refugee camp'/exp OR 'military phenomena'/de OR 'warfare'/de OR 'terrorism'/de OR 'genocide'/de OR 'refugee'/de OR 'forced migrant'/exp OR ‘armed conflict*’:ti,ab,kw
OR ‘War’:ti,ab,kw
OR ‘wars’:ti,ab,kw
OR ‘Internally Displaced Persons’:ti,ab,kw
OR ‘displaced people’:ti,ab,kw
OR ‘refugee*’:ti,ab,kw
OR ‘Genocide’:ti,ab,kw
OR ‘Terrorism’:ti,ab,kw

Climate Change

'natural disaster'/exp OR 'climate change'/exp OR 'greenhouse effect'/exp OR 'climate'/de OR 'environmental impact'/exp OR 'water insecurity'/exp OR 'severe weather'/exp OR ‘Desertification’:ti,ab,kw
OR ‘soil degradation’:ti,ab,kw
OR ‘Climate Change’:ti,ab,kw
OR ‘Global Warming’:ti,ab,kw
OR ‘natural disaster*’:ti,ab,kw
OR ‘drought*’:ti,ab,kw
OR ‘flood*’:ti,ab,kw
OR ‘heat wave*’:ti,ab,kw
OR ‘water scarcity’:ti,ab,kw
OR ‘water shortage*’:ti,ab,kw
OR ‘environmental stress’:ti,ab,kw

Mental Health

'mental health'/exp OR 'anxiety disorder'/exp OR 'anxiety'/de OR 'catastrophizing'/de OR 'posttraumatic stress disorder'/de OR 'depression'/exp OR 'psychological resilience'/exp OR ‘Anxiety’:ti,ab,kw

OR ‘Mental Health’:ti,ab,kw
OR ‘catastrophiz*’:ti,ab,kw
OR ‘PTSD’:ti,ab,kw
OR ‘depression’:ti,ab,kw
OR ‘Psychological Distress’:ti,ab,kw
OR ‘resilien*’:ti,ab,kw
OR ‘coping’:ti,ab,kw
OR ‘psychological trauma’:ti,ab,kw

NOT 'conference abstract'/it

Grey literature on Google

Additional file 1 – Search Strategy

Specific to NGOs: (CHECKED FOR RELEVANCE)

UNDP: wars OR "armed conflict" OR genocide OR terrorism "climate change" OR "global warming" OR "natural disaster" "mental health" OR anxiety OR PTSD OR depression filetype:pdf site:undp.org

wars OR "armed conflict" OR genocide OR terrorism "climate change" OR "global warming" OR "natural disaster" "mental health" OR anxiety OR PTSD OR depression filetype:pdf site:who.int

wars OR "armed conflict" OR genocide OR terrorism "climate change" OR "global warming" OR "natural disaster" "mental health" OR anxiety OR PTSD OR depression filetype:pdf site:unhcr.org

filetype:pdf

(.org, .int) sites with relevant files

("wars" OR "armed conflict" OR "genocide" OR "terrorism") ("climate change" OR "global warming" OR "natural disaster") ("mental health" OR "anxiety" OR "PTSD" OR "depression") site:.org OR site:.int filetype:pdf
