# Supplementary material for: The overlapping effects of climate change and conflict on mental health of vulnerable populations: a scoping review
Source: Confl Health. 2026 Feb 3;20:21. doi: 10.1186/s13031-026-00758-5 (PMC12955018; doi:10.1186/s13031-026-00758-5)
Supplement: Supplementary file 2 — Additional file 2: Data Extraction Form [file 13031_2026_758_MOESM2_ESM.pdf]

Additional File 2: DATA EXTRACTION FORM

Record ID	Citation (Author, Year)	Study design	Region/ Country	Population characteristics	Climate Hazard (IPCC onsets)	War Context	Mental Health Focus	Mechanisms/Pathways	Interventions & Mitigation Strategies Proposed IF ANY	Key insights & Relation to Research Question
21	Logie et al., 2024	Cross-sectional survey as part of a longitudinal study	Kampala Uganda	Refugee youth aged 16-24 77.8% from DRC	Water insecurity/gradual scarcity linked to extreme weather events (slow-onset)	Refugees displaced by armed - conflict residing in Uganda	Depression Anxiety Hopelessness	Extreme weather events --> water scarcity --> water insecurity --> social-ecological stressors (low social support, refugee status, IPV) --> increased depression risk	It suggests that future research can explore strategies to address water insecurity and other social-ecological stressors to promote health and well-being among urban refugee youth. It also mentions that interventions focused on relational, community, and structural-level factors are needed, rather than just intrapersonal ones.	- Water insecurity, longer time in Uganda, lower social support, parenthood, and recent intimate partner violence were associated with moderate depression. - Water insecurity, longer time in Uganda, and lower social support were associated with moderately severe depression. - The Index of Vulnerability (IoV), which combines multiple social-ecological stressors, accounted for more variance in depression among women than any single factor. - For men, water insecurity was the strongest factor associated with moderate depression. - The study highlights the urgency of examining water insecurity and mental health among refugees. - The findings have implications for research and practice aimed at improving urban refugee youth mental health in low- and middle-income countries.
22	Rasmussen, 2020	Qualitative study ethographic	northern Niger and Mali in West Africa.	Tuareg refugees	Drought (slow-onset) floods (rapid-onset)	Regional wars caused the displacement of Tuaregs	Loneliness linked to feelings of solitude, nostalgia, and depression.	Drought + war --> dispersion and displacement --> separation from family + wanting to go home --> loneliness + nostaliga + depression	No proposed interventions but higlights that the Tuareg people cope with loneliness through their cultural practices, narratives, poetry, and songs.	war + drought jointly affect the mental health of Tuareg refugees by dispersing them and separating them from families and homeland
23	Pike, 2004	Case study Semi-longitudinal	Turkana District, Kenya	Ngisonyoka Turkana a nomadic pastoralist group	Droughts (slow-onset)	prolonged conflict between ethnic groups	psychosocial wellbeing distress	Climate change induced drought --> intensify conflict --> the combined effect leads to changes in nomadic traditions --> decline in social wellbeing --> psychosocial stress	population developed resilience and coping strategies no mitigation/interventions suggested	The study highlights how a population's vulnerability to a slow-onset climate disaster, specifically a multi-year drought, is heightened by the pre-existing conditions of armed conflict and violence. It highlights the population's need to adopt new, stressful coping strategies, which in turn enables a decline in psychosocial well-being and leads to a loss of traditional social organization.
24	Ali et al., 2023	Cross sectional survey (Quantitative)	Mogadishu, Somalia	Internally displaced persons (IDPs), majority women	Slow-onset hazards: drought, famine (food/water	Prolonged conflict since 1990	Outcomes: PTSD (32%), depression (59%)	War + climate change --> displacement + trauma --> collapsed health and social systems --> Unemployment, poverty,	-recommends more mental health screening + intervention -no interventions proposed to mitigate climate change though	intersection of conflict and climate-linked famine/drought driving mass displacement and severe mental health burden.

Additional File 2: DATA EXTRACTION FORM

				(83%), ages 18–60+, 64.8% had no formal education, 66.3% unemployed, many displaced multiple times.	scarcity). Rapid-onset (floods)			gendered vulnerabilities --> poor mental well being		
25	Straight et al., 2025	mixed-methods design	Kenya	Youth (M&F) from two different climate zones Mentions pastoralist children	Drought (Slow-onset)	State sponsored violence also violence between Samburu, Pokot, and Turkana over pasture and water points.	drought compounded daily stressors, psychosocial distress, PTSD symptoms	Drought + State violence --> compounded stressors (food and water insecurity, loss of livelihood, violence, destruction of assets) --> psychosocial and physiological distress	QUOTE "Community-driven interventions are needed to reduce precarity for young people pursuing pastoralist livelihoods"	In relation to the VPCM, age and gender are predisposing factors to vulnerability, with girls being more vulnerable. The breakdown of support system (through shooting cattle, and violence) enables the sustainment of this vulnerability in youth. Youth experience siginificat distress, and girls in particular endorsed more PTSD symptoms. Highlighting the need for holistic interventions that address both psychological well-being and the physiological impacts of stress. Resource scarcity a major contributor to conflict and vulnerability.
26	Ecks, 2025	Qualitative study	Myanmar (formerly Burma)	General population affected by war with reference to historical data on refugee and expatriate groups	Natural disasters like cyclones and floods (rapid-onset)	Decades of military dictatorship civil wars religious violence interethnic conflict political oppression government brutality	Focused on depression but also mentions: anxiety mood disorders psychosis trauma mental distress substance use disorders	Climate related disasters + Conflict --> Displacement --> Resource scarcity + military oppression --> worsened mental health	- The author advocates for the collection of accurate, unbiased mental health data, free from political interference. - Educate and support local healthcare providers to recognize and address mood disorders, also reduce the stigma and fear associated with seeking mental health treatment.	- This paper provides a unique perspective on the intersection of conflict and mental health by showing how a political regime can actively suppress the diagnosis and treatment of a mental health condition. - It argues that official statistics showing low rates of depression are not due to a lack of mental illness but rather the result of a deliberate political strategy. - This is a crucial insight for your scoping review, as it demonstrates that in some conflict settings, the reported mental health burden may be an underestimate due to political interference and a lack of medicalization, rather than a true reflection of population health. - The study highlights the complex relationship between war, political control, and healthcare infrastructure in shaping mental health outcomes and the response to them.
27	Trummer et al., 2023	Qualitative study using webinar discussions	Africa with case studies from	migrant and refugee communities, reported	Cyclone Idai (2019) and floods; rapid-onset. Temperature rise,	Arab-Fur war in Sudan (1987-1989)	Traumatic experiences PTSD and anxiety	Climate change leads to resource scarcity --> conflicts over resources --> forced displacement --> health impacts and	Policy recommendations: collaborative efforts between developed/developing countries, interdisciplinary research, evidence-building for	Experts in this webinar confirm the connection between climate change, forced-migration, and health (including mental health), with climate change exacerbating existing vulnerabilities and creating new health challenges for mobile

Additional File 2: DATA EXTRACTION FORM

			Sudan (Darfur/ Kordofan) and Zimbabwe (Chimanimani/Chipinge)	by 25 experts	desertification, droughts; slow-onset			limited healthcare access.	climate governance, mainstreaming climate change into national development policies, international protection for climate-displaced populations. Country examples: Zimbabwe's NDC revision, Rwanda's 38% emission cuts by 2030, Uganda's NDC Partnership Plan (2018-2020)	populations. Need for legitimate international protection claims for populations displaced by climate change they did not cause.
28	Tadesse et al., 2025	mixed-methods design	Oromia region of Ethiopia	Primary caregivers of orphans and vulnerable children Ages ranged 46–67; mostly female, with large families, low education, unstable income, and reliance on NGO support.	Drought, food insecurity, climate variability (slow-onset)	ethnic conflict, internal displacement, external migration, war	well-being stress trauma	Local conflict + drought --> economic strain and food insecurity --> displacement --> stressed caregivers + OVC mental health burden	Suggests economic support and strengthening social networks	The paper provides emperical evidence that caregivers of OVCs in Ethiopia experience compounded stress from both climate hazards (e.g., drought, food insecurity) and ongoing conflict, highlighting a joint effect on mental health.
29	Lindvall et al., 2020	Qualitative interviews	Horn of Africa, specifically Somalia, Kenya, and Ethiopia.	internally displaced persons (IDPs), refugees, and pastoralists.	Droughts (slow-onset) floods mentioned as well (rapid-onset)	Reference to conflict in Somalia as driver of migration/trauma	Trauma in Somali refugees poor mental health in Ethiopians	War + drought --> displacement --> loss of livelihood + GBV + limited access to care --> trauma and poor mental health	- needs assessment + evaluation of mental health needs - One important note "donor funding tends to favor "life-saving" interventions over crucial services like mental health support"	The paper shows that both War and Climate change act as dual drivers of displacement which is a huge source of trauma in vulnerable populations The populations vulnerability is higjlighted in the loss of livelihoods either due to overcrowding, lack of resources, or lack of access to healthcare
30	Igreja, 2003	Exploratory study (mixed methods)	Central Mozambique	women with malnourish	Drought induced famine (slow-onset)	Former warzone (16 years of	psychological health of mothers and the	Prolonged war + drought --> trauma + displacement --> cultural disruptions -->	recommends desiging culturaly sensitive strategies to address the cultural disrpution	The study highlights how a population’s vulnerability to climate disasters, specifically drought and famine, is heightened by pre-existing conditions of war and conflict. This dual

Additional File 2: DATA EXTRACTION FORM

				ed children & elders		armed conflict)	developme nt of children	less social cohesion + diminished resilience --> decline in psychological well being of mothers --> infant malnutrition		vulnerability leads to a breakdown of a population's resilience which in turn enables a decline in mental health outcomes and increases the risk of malnutrition and psychosocial suffering for mothers and children.
31	Marzouk et al., 2022	Cross-sectional survey	Iraq IDP camps in the governo rates of Ninewa and Duhok	Internally displaced persons in Iraq	Heatwaves (rapid -onset) dust storms (rapid-onset) drought (slow-onset)	DPs who were displaced due to decades of internal conflicts and the ISIL military offensive.	psychosocia l wellbeing	Conflict --> Displacement + Climate change hazards (heat, dust storm, drought) --> Poor living conditions (loss of livelihood) --> psychosocial distress <--> lack of access to mental health support	- Educate communities about the causes and consequences of climate change through traditional and social media, awareness sessions, and community involvement in decision-making. - Involve communities in the decision-making processes for climate-related interventions. - government should build shelters and increase access to clean water to help IDPs cope with the impacts of extreme weather conditions.	The study shows that most IDPs have observed and been directly affected by climate change. However, many do not view addressing climate change as a priority, prioritizing their basic needs due to conflict and displacement. The research shows that climate change adds to the vulnerability of people already displaced by conflict.
32	Devonald et al., 2022	Qualitative study with conceptual framing	Lebano n	Adolescents and youth (ages 16-25) in refugee and host communitie s, including Syrians and Palestinians	Water scarcity pollution high temperatures (slow-onset)	Refigees from Syria and Palestine displaced due to The Syrian revolution The Nakba + Israeli Invasion of Palestine	Psychosocia l wellbeing hopelessne ss depression	War + conflict --> displacement --> economic crisis in host country --> increased unaddressed climate stressors --> poor sanitation, water scarcity, and pollution --> enviromental & economic challeneges (no jobs + inadequate housing + high cost of living) --> decline in psycosocial wellbeing	The authors propose a more integrated approach to sustainable development. They outline measures such as investing in skills for the "green economy," adolescent-friendly social protection, and improved shelter, health, and WASH facilities to respond to the impacts of climate change.	The study highlights how a population's vulnerability to climate disasters, specifically water scarcity and pollution, is heightened by pre-existing conditions of forced displacement and economic crisis. This dual vulnerability leads to a breakdown of a population's resilience, as seen in inadequate housing and the lack of social cohesion, which in turn enables a decline in mental health outcomes, such as depression and hopelessness, among adolescents.
33	Kim et al., 2007	Cross-sectional survey	South Darfur Sudan	Internally displaced persons (IDPs), with a focus on women	Drought, desertification (slow onset)	Armed conflict in Darfur leading to displacement into camps	Psychologic al distress, trauma-related symptoms, depression, and anxiety	War in Darfur --> mass displacement + violence drought --> food/water insecurity + resource depletion jointly (war+climate) --> compounded stressors of displacement --> poor mental health outcomes	- Strengthening women's health and psychosocial support services in IDP camps - Improving access to basic needs (food, water, health care)	The study highlights how conflict-driven displacement interacts with climate stressors (drought, desertification, food scarcity) to worsen women's mental health in IDP camps. It underscores the interlinked vulnerabilities created by war and climate hazards

Additional File 2: DATA EXTRACTION FORM

34	Albahsahli et al., 2023	Preprint mixed-methods study	San Diego, California, USA	Arabic-speaking refugees from Iraq and Syria	Extreme cold in Turkey Extreme heat in Jordan Dust storm in Jordan (rapid-onset)	Conflict and persecution in Syria and Iraq	Perceived mental health	war (trauma +stress) --> displacement + climate stressors (heat/dust) --> poorer mental health --> compounding stress --> chronic health inequity	The paper aims to inform policy that closes the gap in climate health of refugees in general. No intervention or mitigation strategies suggested	- The study found a link between exposure to poor weather conditions and poorer mental health - It reveals that refugees are highly vulnerable to climate-sensitive exposures but often do not perceive a direct link between these exposures and their physical health. Instead, they attributed physical health issues to the stress of war.
35	Hall et al., 2025	Cluster-randomized controlled trial (cRCT) study protocol	Uganda (Nakivale refugee settlement)	Refugee mothers and children (36–59 months)	Food insecurity due to climate change (slow-onset)	Refugee population focibly displaced due to conflict	Psychological distress PTSD Depression Wellbeing	Pre-existing vulnerability due to displacement, trauma, and loss --> Increased Exposure to Climate Hazards (e.g., Water insecurity, agricultural disruption) --> Psychological Distress (e.g., Depression, anxiety, PTSD, hopelessness) --> Inability to Adapt --> Continued Cycle of Hardship	3 Interventions being tested: Enhanced Usual Care (EUC): A psychoeducation session and information on existing mental health services. Home Gardening Intervention (HGI): Provides agricultural inputs and training for climate-resilient farming to improve food security. HGI/SH+: Combines the Home Gardening Intervention with Self-Help Plus (SH+), a group-based mental health intervention based on Acceptance and Commitment Therapy.	Ongoing intervention trial that provides a framework for how mental health can be both an outcome of these combined stressors and a crucial factor in building resilience and improving adaptive capacity. It directly tests the hypothesis that addressing mental health can serve as a mitigation strategy for climate-related food insecurity in a conflict-affected population.
36	Azhar et al., 2023	Letter to the Editor conceptual commentary	Myanmar (also referred to as Burma). Also references Afghanistan, Azerbaijan, and Nepal as comparative examples	Specifically mentions women, children, pregnant women, and internally displaced people (IDPs) including the Rohingya, Kachin, Shan, Karens, and Rakhine ethnic groups.	Cyclones (rapid-onset)	Ongoing military conflict in Myanmar between the State Administration Council and the National Unity Government. It also references a history of ethnic and religious discrimination and civil conflict dating back to World War II	Mental well-being psychological distress depression anxiety PTSD	Vulnerability from civil war and ethnic discrimination --> Rapid-Onset Climate Hazard --> Combined Trauma & Psychological Distress --> Increased prevalence of anxiety, depression, and PTSD --> Barriers to Humanitarian Aid & Recovery (Political interference, discrimination, stigma, lack of infrastructure) --> Continued Psychosocial Stress	- Implement a comprehensive mental health and psychosocial support (MHPSS) program. - Educate community leaders on psychological care skills. - Train and supervise primary healthcare workers in counseling and psychotropic medication. - Ensure timely and reliable communication to the public and uninterrupted access to medication for psychiatric patients. - Deploy tele-psychiatry, which has been successful in Bangladesh and Syria. - Integrate psychosocial support into hospitals and educational institutions. - Promote mental health awareness and use volunteers with lived experience to provide support. - Establish a strong community mental health	- The study shows a clear intersection of war and climate change, with a rapid-onset climate event (Cyclone Mocha) compounding the mental health challenges already present from ongoing civil war and displacement. - It highlights how political factors and government responses, such as delaying aid and discrimination, can worsen mental health outcomes and hinder recovery efforts in a post-disaster setting. - The article argues for the urgent need to integrate mental health and psychosocial support (MHPSS) programs into disaster response, as they are often overlooked in favor of immediate needs like food and shelter. - It emphasizes the importance of community-based approaches, like educating leaders and using volunteers with lived experience, to combat the stigma surrounding mental health and ensure effective care.

Additional File 2: DATA EXTRACTION FORM

									system as a form of disaster preparedness.	
37	Asad et al., 2013	Conceptual framework	Pakistan	Internally displaced children and their families living in camps.	Flood (rapid-onset)	Populations displaced due to a decade-long conflict in the Northern provinces along the border" and "military operations against militants	General mental health mentions PTSD & depression	War + floods --> displacement Inhumane living conditions --> vulnerability and mental health decline --> child trauma & abuse	Psychosocial framework interventions: - community-based recreational and cultural activities for children. - education & awareness - group therapy for adults and children	The paper clearly presents the intersection of climate change and conflict causing displacement, which creates conditions that significantly increase the risk of abuse, trauma, and other negative mental health outcomes for children. The paper also highlights how the combined effects of these two drivers of displacement create a vulnerable population living in an environment that lacks resources and support, leading to serious shortcomings in social, emotional, and cognitive development.
38	Eboreime et al., 2025	Perspective with conceptual framing	West African Sahel region Burkina Faso Nigeria and others	Internally displaced persons (IDPs) and refugees in the region	Rising temperatures inconsistent rainfall floods (rapid-onset) desertification + droughts (slow-onset)	ongoing violent conflict and forced migration in the region. violent clashes due to competition over scarce resources such as (Boko haram + Jihadists)	General psychological wellbeing Depression PTSD Suicide Substance abuse	climate shifts (rising temperatures, reduced rainfall, desertification) --> resource scarcity (water and land) --> intensified conflict between communities --> forced displacement --> psychosocial health impacts	The paper calls for: - Improving climate resilience through climate-smart agriculture and water resource protection. - Expanding physical, food, and psychosocial assistance programs tailored to the complex needs of displaced groups. - Strengthening human security and gender-based violence prevention in IDP camps. - Improving data collection and establishing coordinated regional strategies for climate-displaced populations.	- climate change is a "threat multiplier" for conflict and displacement in West Africa. - The paper explicitly links forced migration and the conditions in IDP camps exposed to climaete hazards to severe psychosocial health impacts, including high rates of depression, anxiety, and PTSD.
39	Maukera & Blignault, 2015	Literature review	The Solomon Islands	General population of Solomon Island	Cyclones + heavy rains + flash floods (rapid-onset) Sealevel rise (slow-onset)	war from 1998 to 2003 known locally as "the tensions"	mental health and psychosocial well-being mentions major depressive disorder suicide substance abuse	war + climate hazards --> affected livliehood (homelessness) --> trauma + mental decline --> long term behavioral changes --> violence + substance abuse + self-harm	National Mental Health Strategy, which focuses on prevention, promotion, and early intervention. It also notes the need for continued investment in mental health services and the importance of professional development and training for Solomon Islands mental health professionals.	The study highlights how a population's vulnerability to rapid- and slow-onset climate disasters is heightened by the pre-existing conditions of armed conflict and political unrest. This dual vulnerability leads to a breakdown of a population's resilience, as seen in the lingering fear and disruption of social cohesion, which in turn enables a decline in mental health outcomes, such as depression, substance abuse, and interpersonal violence.

Additional File 2: DATA EXTRACTION FORM

40	Zafar et al., 2016	Peer reviewed clinical trial protocol	Swat district in Northern Pakistan	The trial targets women with perinatal depression, but aims to train 80 Lady Health Workers (LHWs), who are community health workers providing preventive primary care services.	2010 Flood (rapid-onset)	Post-conflict area Taliban resurgence Military operations	Perinatal depression	War (displacement, violence, damage) + Floods (infrastructure damage, loss of homes) --> humanitarian crisis --> cumulative trauma --> increased risk of depression --> increased perinatal depression in women	2 interventions: 1- Technology-assisted Cascade Training and Supervision (TACTS), which uses a tablet-based manual for training and supervision. 2- control group receiving specialist-delivered training directly from mental health experts.	This protocol offers a response to the humanitarian crisis caused by both conflict and devastating flood in Pakistan. The proposed interventions aim o address the mental health of a vulnerable population (mothers), which also directly addresses the health of their offsprings.
41	Syed Sherif et al., 2011	Review Article (country profile)	Somalia	General pop of Somalia, of whom 65% are nomadic. mentions women, and those who seek traditional healers as well as IDPs	Drought (Slow-onset)	Violece and civil war especially in the south	General overveiw of mental health	Drought + Political violence --> displacement + resource scarcity --> trauma + loss --> compounding mental health impacts	Lack of research and faculty studying mental health in Somalia mental health is taught to medical students in their last year, and internships in collaboration with UK colleges to educate healthcare professionals about mental health	The paper provides an overview of the state of mental healtcare in Somalia, a country affected by both climate hazard (drought) and civil war. With a large number of IDPs and refugees, so little awareness of mental health, and the reliance on traditional/religious healers exacerbates the mental health impacts.
42	Schuster et al., 2024	Perspective with conceptual framing	Bangladesh, specifically Cox bazar (the world's largest refugee camp)	Forcibly displaced populations, with a specific focus on Rohingya women displaced in Bangladesh	Fire (rapid-onset) Lamdslide (rapid-onset) Direcr results of climate chage = water and food insecurity	displacement fleeing persecution in Burma/Myanmar	Mental distress also mentions resilience	Displacement due to conflict + Climate hazards --> humanitarian crisis in refugee camp --> gendered inequities --> excacerbated mental health issues in women	fostering resilience through technology: - sharing health information - combating misinformation - early warning systems for fires - strengthening community resources	The paper presents a framework where the initial forced displacement caused by persecution in Burma/Myanmar creates a baseline of vulnerability, and then climate hazards like fires, landslides, and food/water insecurity act as compounding stressors. This combined effect exacerbates existing health inequities and contributes to a humanitarian crisis.

Additional File 2: DATA EXTRACTION FORM

43	Sanni et al., 2022	Scoping review of 7 records (3 cross-sectional, 2 qualitative, 1 cohort, and 1 need assessment report)	Nigeria, Kenya, Tanzania, Somalia, and the USA	African immigrant; refugees, internally displaced persons (IDPs), and rural-to-urban migrants	Flooding Drought Excess heat slow and rapid onsets	One included study mentions Somali civil war in Africa causing forced displacement	Poor mental health distress trauma	War + Climate hazards --> forced displacement --> loss of social capital --> poor mental health	The paper proposes the following : Governments should develop a long-term strategy to prevent or mitigate the health-related effects of natural disasters, which would go beyond just a rapid-response system. It also highlights the "stark need for mental health support services" for migrants and internally displaced persons, who often face traumatic conditions due to climate change.	in this paper climate change is described as a "threat multiplier" that can increase the risk of conflict. Both war and climate change, through events like drought, can force people to leave their homes, which is a traumatic experience. This displacement and the related trauma, along with the loss of social networks, contribute to mental health issues such as stress, sadness, loneliness, and emotional disorders
44	Ae-Ngibise et al., 2021	Systematic review of 7 records six of which used a cross-sectional design and one an experimental design.	West Africa, with five of the seven included studies based in Ghana.	children and adults	Drought (slow-onset)	Displacement due to political and civil security instability caused by insurgent groups	psychological distress, anxiety, sleeplessness	Drought + WAR --> Food insecurity --> poor mental health	increasing food supply and providing mental health interventions for all ages.	The review shows a strong association between food insecurity and mental health outcomes, and highlights that food insecurity is often caused by both climate (drought) and war, also displacement plays a huge role
45	McMichael, 2014	Review Article	Global, with specific examples from Somalia and Kenya	Focused on children	Reference to Drought in Somalia (Slow-onset)	Civil war in Somalia + other resource conflicts	Chronic anxiety, worry, and emotional disorders	climate driven drought + monsoon + Indian ocean warming + chronic war+displacement --> "camps are now filled to four times their planned capacity, 50% are children (vulnerable) --> direct risk of mental/emotional impacts + trauma Also mentions direct effect of children observing their families stressed abotu climate change	No inteventions, just a conceptual framing and advocacy - educating children on ecological understanding can help hem become agents of change. -	children are a uniquely vulnerable population to the joint effects of climate change and war. The intersection of a climate hazard (drought) and conflict (civil war) is shown to create a compounded crisis of displacement, which directly leads to trauma and mental and physical health issues.
46	Weissbecker, 2009	Narrative review	Global perspective, mention	General populations, with emphasis	Natural disasters: (slow/rapid-	Civil wars and conflicts in: - Liberia, Sierra Leone, and	PTSD, depression, anxiety, substance	War/Natural disasters --> Displacement --> disrupted services, chronic stress --> Mental	- Human rights–based approaches (ICESCR, CRC, CRPD) - Community-based mental health care - Integration of	- This paper explicitly highlights that both disasters (linked to climate hazards) and wars independently and jointly contribute to severe, long-term mental health burdens. - Vulnerable

Additional File 2: DATA EXTRACTION FORM

		conceptual framing	s LMICs (e.g., Sudan, Sri Lanka, Afghanistan, Liberia, Sierra Leone, Rwanda)	on vulnerable groups (refugees, women, children, elderly, disabled, those with pre-existing mental health problems)	onsets) mentions: drought flood	Rwanda (post-genocide and civil war recovery) - Sri Lanka (post-conflict, following civil war and tsunami recovery) - Sudan (Darfur and South Sudan conflict settings) - Afghanistan (chronic war and instability)	abuse, suicidality, long-term psychosocial impairment, adjustment difficulties	Health Outcomes: (PTSD, depression, anxiety, psychosocial distress)	mental health into primary care (mhGAP) - IASC Guidelines on Mental Health and Psychosocial Support in Emergency Settings - Policies/legislation protecting rights of conflict/disaster-affected persons	groups are at higher risk of persistent psychological distress. - Mental health is framed as a human right, underscoring the importance of integrating psychosocial support into humanitarian response and recovery.
47	Javed, 2016	Book chapter conceptual framing	Pakistan Kashmir and North West Pakistan and the coastal regions.	Populations affected by disasters and fleeing war in Afghanistan with specific focus on children, women, and older adults.	Natural disasters: (rapid-onset) floods landslides cyclones	People fleeing the war in Afghanistan	psychological and psychosocial adversities	War --> displacement --> vulnerable conditions --> climate hazards worsen conditions --> loss of livelihood (lack of resources/loss of social capital/ scarcity of mental health services) --> trauma	Natural Disaster Management Authority (NDMA). It argues for ongoing measures, a consolidated policy, and strengthening institutions to tackle these challenges.	The chapter highlights that Pakistan is a region vulnerable to both natural disasters and war, both of which lead to trauma and forced displacement, causing long-lasting psychosocial problems. These impacts are made worse by the scarcity of mental health services in the country, making it difficult to cope with the "continuous and ongoing war and terrorism".
