# Supplementary material for: The overlapping effects of climate change and conflict on mental health of vulnerable populations: a scoping review
Source: Confl Health. 2026 Feb 3;20:21. doi: 10.1186/s13031-026-00758-5 (PMC12955018; doi:10.1186/s13031-026-00758-5)
Supplement: Supplementary file 4 — Additional file 4: Table 2- Study Characterstics of Included Articles [file 13031_2026_758_MOESM4_ESM.pdf]

Table 2: Study Characteristics of Included Articles

Record ID	Citation (Author, Year)	Study design	Region/Country	Population characteristics	Climate Hazard (IPCC onsets)	War Context	Mental Health Focus
[21]	Logie et al., 2024	Cross-sectional survey as part of a longitudinal study Empirical	Kampala Uganda	Refugee youth aged 16-24 77.8% from DRC	Water insecurity/gradual scarcity linked to extreme weather events (slow-onset)	Refugees displaced by armed -conflict residing in Uganda	Depression Anxiety Hopelessness
[22]	Rasmussen, 2020	Qualitative study ethographic Empirical	northern Niger and Mali in West Africa.	Tuareg refugees	Drought (slow-onset) floods (rapid-onset)	Regional wars caused the displacement of Tuaregs	Loneliness linked to feelings of solitude, nostalgia, and depression.
[23]	Pike, 2004	Case study Semi-longitudinal Empirical	Turkana District, Kenya	Ngisonyoka Turkana a nomadic	Droughts (slow-onset)	prolonged conflict between ethnic groups	psychosocial wellbeing distress

Table 2: Study Characteristics of Included Articles

Record ID	Citation (Author, Year)	Study design	Region/Country	Population characteristics	Climate Hazard (IPCC onsets)	War Context	Mental Health Focus
				pastoralist group			
[24]	Ali et al., 2023	Cross sectional survey (Quantitative) Empirical	Mogadishu, Somalia	Internally displaced persons (IDPs), majority women (83%), ages 18–60+, 64.8% had no formal education, 66.3% unemployed, many displaced multiple times.	Slow-onset hazards: drought, famine (food/water scarcity). Rapid-onset (floods)	Prolonged conflict since 1990	Outcomes: PTSD (32%), depression (59%)
[25]	Straight et al., 2025	mixed-methods	Kenya	Youth (M&F) from two different	Drought (Slow-onset)	State sponsored violence also violence between	drought compounded daily

Table 2: Study Characteristics of Included Articles

Record ID	Citation (Author, Year)	Study design	Region/Country	Population characteristics	Climate Hazard (IPCC onsets)	War Context	Mental Health Focus
		design Empirical		climate zones Mentions pastoralist children		Samburu, Pokot, and Turkana over pasture and water points.	stressors, psychosocial distress, PTSD symptoms
[26]	Ecks, 2025	Qualitative study Empirical	Myanmar (formerly Burma)	General population affected by war with reference to historical data on refugee and expatriate groups	Natural disasters like cyclones and floods Drought (rapid-onset)	Decades of military dictatorship civil wars religious violence interethnic conflict political oppression government brutality	Focused on depression but also mentions: anxiety mood disorders psychosis trauma mental distress substance use disorders

Table 2: Study Characteristics of Included Articles

Record ID	Citation (Author, Year)	Study design	Region/Country	Population characteristics	Climate Hazard (IPCC onsets)	War Context	Mental Health Focus
[27]	Trummer et al., 2023	Qualitative study using webinar discussions Empirical	Africa with case studies from Sudan (Darfur/Kordofan) and Zimbabwe (Chimanimani/Chipinge)	migrant and refugee communities, reported by 25 experts	Cyclone Idai (2019) and floods; rapid-onset. Temperature rise, desertification, droughts; slow-onset	Arab-Fur war in Sudan (1987-1989)	Traumatic experiences PTSD and anxiety
[28]	Tadesse et al., 2025	mixed-methods design Empirical	Oromia region of Ethiopia	Primary caregivers of orphans and vulnerable children Ages ranged 46–67; mostly female, with large families, low education, unstable income, and	Drought, food insecurity, climate variability (slow-onset)	ethnic conflict, internal displacement, external migration, war	well-being stress trauma

Table 2: Study Characteristics of Included Articles

Record ID	Citation (Author, Year)	Study design	Region/Country	Population characteristics	Climate Hazard (IPCC onsets)	War Context	Mental Health Focus
				reliance on NGO support.			
[29]	Lindvall et al., 2020	Qualitative interviews Empirical	Horn of Africa, specifically Somalia, Kenya, and Ethiopia.	internally displaced persons (IDPs), refugees, and pastoralists.	Droughts (slow-onset) floods mentioned as well (rapid-onset)	Reference to conflict in Somalia as driver of migration/trauma	Trauma in Somali refugees poor mental health in Ethiopians
[30]	Igreja, 2003	Exploratory study (mixed methods) Empirical	Central Mozambique	women with malnourished children & elders	Drought induced famine (slow-onset)	Former warzone (16 years of armed conflict)	psychological health of mothers and the development of children trauma from torture, sexual violence, etc

Table 2: Study Characteristics of Included Articles

Record ID	Citation (Author, Year)	Study design	Region/Country	Population characteristics	Climate Hazard (IPCC onsets)	War Context	Mental Health Focus
[31]	Marzouk et al., 2022	Cross-sectional survey Empirical	Iraq IDP camps in the governorates of Ninewa and Duhok	Internally displaced persons in Iraq	Heatwaves (rapid -onset) dust storms (rapid-onset) drought (slow-onset)	DPs who were displaced due to decades of internal conflicts and the ISIL military offensive.	psychosocial wellbeing
[32]	Devonald et al., 2022	Qualitative study with Empirical	Lebanon	Adolescents and youth (ages 16-25) in refugee and host communities, including Syrians and Palestinians	Water scarcity pollution high temperatures (slow-onset)	Refugees from Syria and Palestine displaced due to The Syrian revolution The Nakba + Israeli Invasion of Palestine	Psychosocial wellbeing hopelessness depression adolescent anxiety and depression
[33]	Kim et al., 2007	Cross-sectional	Darfur Sudan	Internally displaced persons (IDPs),	Drought, desertification (slow onset)	Armed conflict in Darfur leading to	Psychological distress, trauma-related

Table 2: Study Characteristics of Included Articles

Record ID	Citation (Author, Year)	Study design	Region/Country	Population characteristics	Climate Hazard (IPCC onsets)	War Context	Mental Health Focus
		survey Empirical		with a focus on women		displacement into camps	symptoms, depression, and anxiety
[34]	Albahsahli et al., 2023	Preprint mixed-methods study Empirical	San Diego, California, USA Climate hazards reported in Jordan & Turkey	Arabic-speaking refugees from Iraq and Syria	Extreme cold in Turkey Extreme heat in Jordan Dust storm in Jordan (rapid-onset)	Conflict and persecution in Syria and Iraq	Perceived mental health
[35]	Hall et al., 2025	Cluster-randomized controlled trial (cRCT) study protocol Conceptual	Uganda (Nakivale refugee settlement)	Refugee mothers and children (36–59 months)	Food insecurity due to climate change (slow-onset)	Refugee population forcibly displaced due to conflict	Psychological distress PTSD Depression Wellbeing

Table 2: Study Characteristics of Included Articles

Record ID	Citation (Author, Year)	Study design	Region/Country	Population characteristics	Climate Hazard (IPCC onsets)	War Context	Mental Health Focus
[36]	Azhar et al., 2023	Letter to the Editor conceptual commentary	Myanmar (also referred to as Burma). Also references Afghanistan, Azerbaijan, and Nepal as comparative examples	Specifically mentions women, children, pregnant women, and internally displaced people (IDPs) including the Rohingya, Kachin, Shan, Karens, and Rakhine ethnic groups.	Cyclones (rapid-onset)	Ongoing military conflict in Myanmar between the State Administration Council and the National Unity Government. It also references a history of ethnic and religious discrimination and civil conflict dating back to World War II	Mental well-being psychological distress depression anxiety PTSD perinatal mental health and approaches to psychosocial care incl. telehealth
[37]	Asad et al., 2013	Conceptual framework	Pakistan	Internally displaced children and	Flood (rapid-onset)	Populations displaced due to a decade-long conflict in the Northern	General mental health mentions PTSD &

Table 2: Study Characteristics of Included Articles

Record ID	Citation (Author, Year)	Study design	Region/Country	Population characteristics	Climate Hazard (IPCC onsets)	War Context	Mental Health Focus
				their families living in camps.		provinces along the border" and "military operations against militants	depression trauma & abuse in minors
[38]	Eboreime et al., 2025	Perspective with conceptual framing	West African Sahel region Burkina Faso Nigeria	Internally displaced persons (IDPs) and refugees in the region	Rising temperatures inconsistent rainfall floods (rapid-onset) desertification + droughts (slow-onset)	ongoing violent conflict and forced migration in the region. violent clashes due to competition over scarce resources such as (Boko haram + Jihadists)	General psychological wellbeing Depression PTSD Suicide Substance abuse psychiatric emergencies from heat and food insecurity

Table 2: Study Characteristics of Included Articles

Record ID	Citation (Author, Year)	Study design	Region/Country	Population characteristics	Climate Hazard (IPCC onsets)	War Context	Mental Health Focus
[39]	Maukera & Blignault, 2015	Literature review Conceptual	The Solomon Islands	General population of Solomon Island	Cyclones + heavy rains + flash floods (rapid-onset) Sea-level rise (slow-onset)	war from 1998 to 2003 known locally as "the tensions"	mental health and psychosocial well-being mention major depressive disorder suicide substance abuse
[40]	Zafar et al., 2016	Peer reviewed clinical trial protocol Conceptual (no data)	Swat district in Northern Pakistan	The trial targets women with perinatal depression, but aims to train 80 Lady Health Workers (LHWs), who are community health workers	2010 Flood (rapid-onset)	Post-conflict area Taliban resurgence Military operations	Perinatal depression

Table 2: Study Characteristics of Included Articles

Record ID	Citation (Author, Year)	Study design	Region/Country	Population characteristics	Climate Hazard (IPCC onsets)	War Context	Mental Health Focus
				providing preventive primary care services.			
[41]	Syed Sherif et al., 2011	Review Article (country profile) Conceptual	Somalia	General pop of Somalia, of whom 65% are nomadic. mentions women, and those who seek traditional healers as well as IDPs	Drought (Slow-onset)	Violence and civil war especially in the south	General overview of mental health
[42]	Schuster et al., 2024	Perspective with conceptual framing	Bangladesh, specifically Cox bazar (the world's largest refugee camp)	Forcibly displaced populations, with a specific focus on	Fire (rapid-onset) Landslide (rapid-onset) Direct results of climate change = water	displacement fleeing persecution in Burma/Myanmar	Mental distress also mentions resilience

Table 2: Study Characteristics of Included Articles

Record ID	Citation (Author, Year)	Study design	Region/Country	Population characteristics	Climate Hazard (IPCC onsets)	War Context	Mental Health Focus
				Rohingya women displaced in Bangladesh	and food insecurity		
[43]	Sanni et al., 2022	Scoping review Conceptual	Nigeria, Kenya, Tanzania, Somalia	African immigrant; refugees, internally displaced persons (IDPs), and rural-to-urban migrants	Flooding Drought Excess heat slow and rapid onsets	One included study mentions Somali civil war in Africa causing forced displacement	Poor mental health distress trauma
[44]	Ae-Ngibise et al., 2021	Systematic review Conceptual	West Africa, with five of the seven included studies based in Ghana.	children and adults	Drought (slow-onset)	Displacement due to political and civil security instability caused by insurgent groups	psychological distress, anxiety, sleeplessness

Table 2: Study Characteristics of Included Articles

Record ID	Citation (Author, Year)	Study design	Region/Country	Population characteristics	Climate Hazard (IPCC onsets)	War Context	Mental Health Focus
[45]	McMichael, 2014	Review Article Conceptual	Global, with specific examples from Somalia and Kenya	Focused on children	Reference to Drought in Somalia (Slow-onset)	Civil war in Somalia + other resource conflicts	Chronic anxiety, worry, and emotional disorders
[46]	Weissbecker, 2009	Narrative review conceptual framing	Global perspective, mentions LMICs (specifically Sudan, Sri Lanka, Afghanistan, Liberia, Sierra Leone, Rwanda)	General populations, with emphasis on vulnerable groups (refugees, women, children, elderly, disabled, those with pre-existing mental health problems)	Natural disasters: (slow/rapid-onsets) mentions: drought flood	Civil wars and conflicts in: - Liberia, Sierra Leone, and Rwanda (post-genocide and civil war recovery) - Sri Lanka (post-conflict, following civil war and tsunami recovery) - Sudan (Darfur and South Sudan conflict settings) - Afghanistan	PTSD, depression, anxiety, substance abuse, suicidality, long-term psychosocial impairment, adjustment difficulties

Table 2: Study Characteristics of Included Articles

Record ID	Citation (Author, Year)	Study design	Region/Country	Population characteristics	Climate Hazard (IPCC onsets)	War Context	Mental Health Focus
						(chronic war and instability)	
[47]	Javed, 2016	Book chapter conceptual framing	Pakistan Kashmir and North West Pakistan and the coastal regions.	Populations affected by disasters and fleeing war in Afghanistan with specific focus on children, women, and older adults.	Natural disasters: (rapid-onset) floods landslides cyclones	People fleeing the war in Afghanistan	psychological and psychosocial adversities
