# Supplementary material for: The overlapping effects of climate change and conflict on mental health of vulnerable populations: a scoping review
Source: Confl Health. 2026 Feb 3;20:21. doi: 10.1186/s13031-026-00758-5 (PMC12955018; doi:10.1186/s13031-026-00758-5)
Supplement: Supplementary file 5 — Additional file 5: Table 3- Pathways of Interaction [file 13031_2026_758_MOESM5_ESM.pdf]

Table 3: Pathways of interaction between Climate Change and War and their Effects on Mental Health

Record ID	Citation (Author, Year)	Mechanisms/Pathways	Key insights & Relation to Research Question
[21]	Logie et al., 2024	Extreme weather events<br>--> water scarcity --> water insecurity<br>--> social-ecological stressors (low social support, refugee status, IPV)<br>--> increased depression risk	- Water insecurity, longer time in Uganda, lower social support, parenthood, and recent intimate partner violence were associated with moderate depression.<br>- Water insecurity, longer time in Uganda, and lower social support were associated with moderately severe depression.<br>- The Index of Vulnerability (IoV), which combines multiple social-ecological stressors, accounted for more variance in depression among women than any single factor.<br>- For men, water insecurity was the strongest factor associated with moderate depression.<br>- The study highlights the urgency of examining water insecurity and mental health among refugees.<br>- The findings have implications for research and practice aimed at improving urban refugee youth mental health in low- and middle-income countries.
[22]	Rasmussen, 2020	Drought + war<br>--> dispersion and displacement<br>--> separation from family + wanting to go home<br>--> loneliness + nostalgia + depression	war + drought jointly affect the mental health of Tuareg refugees by dispersing them and separating them from families and homeland
[23]	Pike, 2004	Climate change induced drought<br>--> intensify conflict<br>--> the combined effect leads to changes in nomadic traditions<br>--> decline in social wellbeing<br>--> psychosocial stress	The study highlights how a population’s vulnerability to a slow-onset climate disaster, specifically a multi-year drought, is heightened by the pre-existing conditions of armed conflict and violence.<br>It highlights the population's need to adopt new, stressful coping strategies, which in turn enables a decline in psychosocial well-being and leads to a loss of traditional social organization.

[24]	Ali et al., 2023	War + climate change --> displacement + trauma<br>--> collapsed health and social systems<br>--> Unemployment, poverty, gendered vulnerabilities<br>--> poor mental well being	intersection of conflict and climate-linked famine/drought driving<br>mass displacement and severe mental health burden.
[25]	Straight et al., 2025	Drought + State violence<br>--> compounded stressors<br>(food and water insecurity, loss of livelihood, violence, destruction of assets)<br>--> psychosocial and physiological distress	- In relation to the VPCM, age and gender are predisposing factors to vulnerability, with girls being more vulnerable. The breakdown of support system (through shooting cattle, and violence) enables the sustainment of this vulnerability in youth.<br>Youth experience significant distress, and girls in particular endorsed more PTSD symptoms. Highlighting the need for holistic interventions that address both psychological well-being and the physiological impacts of stress.<br>Resource scarcity a major contributor to conflict and vulnerability.
[26]	Ecks, 2025	Climate related disasters + Conflict<br>--> Displacement<br>--> Resource scarcity + military oppression<br>--> worsened mental health	- This paper provides a unique perspective on the intersection of conflict and mental health by showing<br>how a political regime can actively suppress the diagnosis and treatment of a mental health condition.<br>- It argues that official statistics showing low rates of depression are not due to a lack of mental illness but<br>rather the result of a deliberate political strategy.<br>- This is a crucial insight for your scoping review, as it demonstrates that in some conflict settings, the reported mental health burden may be an underestimate due to political interference and a lack of medicalization, rather than a true reflection of population health.<br>- The study highlights the complex relationship between war, political control, and healthcare infrastructure in shaping<br>mental health outcomes and the response to them.
[27]	Trummer et al., 2023	Climate change leads to resource scarcity<br>--> conflicts over resources	Experts in this webinar confirm the connection between climate change,

		--> forced displacement<br>--> health impacts and limited healthcare access.	forced-migration, and health (including mental health), with climate change exacerbating existing vulnerabilities and creating new health challenges for mobile populations. Need for legitimate international protection claims for populations<br>displaced by climate change they did not cause.
[28]	Tadesse et al., 2025	Local conflict + drought<br>--> economic strain and food insecurity<br>--> displacement<br>--> stressed caregivers + OVC mental health burden	The paper provides empirical evidence that caregivers of OVCs in Ethiopia experience compounded stress from both climate<br>hazards (e.g., drought, food insecurity) and ongoing conflict, highlighting a joint effect on mental health.
[29]	Lindvall et al., 2020	War + drought --> displacement<br>--> loss of livelihood + GBV<br>+ limited access to care<br>--> trauma and poor mental health	- The paper shows that both War and Climate change act as dual drivers of displacement which is a huge source of trauma<br>in vulnerable populations<br>- The populations vulnerability is highlighted in the loss of livelihoods either due to overcrowding, lack of resources, or lack<br>of access to healthcare
[30]	Igreja, 2003	Prolonged war + drought<br>--> trauma + displacement<br>--> cultural disruptions<br>--> less social cohesion + diminished resilience<br>--> decline in psychological well being of mothers<br>--> infant malnutrition	The study highlights how a population's vulnerability to climate disasters, specifically drought and famine, is heightened by<br>pre-existing conditions of war and conflict. This dual vulnerability leads to a breakdown of a<br>population's resilience which in turn enables a decline in mental health outcomes and increases the risk of malnutrition and<br>psychosocial suffering for mothers and children.
[31]	Marzouk et al., 2022	Conflict --> Displacement + Climate change hazards<br>(heat, dust storm, drought)<br>--> Poor living conditions (loss of livelihood)<br>--> psychosocial distress <--> lack of access to mental health support	The study shows that most IDPs have observed and been directly affected by climate change. However, many<br>do not view addressing climate change as a priority, prioritizing their basic needs due to conflict<br>and displacement. The research shows that climate change adds to the vulnerability of people already displaced by conflict.

[32]	Devonald et al., 2022	War + conflict<br>--> displacement<br>--> economic crisis in host country<br>--> increased unaddressed climate stressors<br>--> poor sanitation, water scarcity, and pollution<br>--> environmental & economic challenges (no jobs + inadequate housing + high cost of living)<br>--> decline in psychosocial wellbeing	The study highlights how a population's vulnerability to climate disasters, specifically water scarcity and pollution, is heightened by pre-existing conditions of forced displacement and economic crisis. This dual vulnerability leads to a breakdown of a population's resilience, as seen in inadequate housing and the lack of social<br>cohesion, which in turn enables a decline in mental health outcomes, such as depression<br>and hopelessness, among adolescents.
[33]	Kim et al., 2007	War in Darfur --> mass displacement + violence<br>drought --> food/water insecurity + resource depletion<br><br>jointly (war+climate) --> compounded stressors of displacement<br>--> poor mental health outcomes	The study highlights how conflict-driven displacement interacts with climate stressors (drought, desertification, food scarcity)<br>to worsen women's mental health in IDP camps. It underscores the interlinked vulnerabilities created<br>by war and climate hazards
[34]	Albahsahli et al., 2023	war (trauma +stress) --> displacement<br>+ climate stressors (heat/dust)<br>--> poorer mental health<br>--> compounding stress<br>--> chronic health inequity	- The study found a link between exposure to poor weather conditions and poorer mental health<br>- It reveals that refugees are highly vulnerable to climate-sensitive exposures but often do not perceive a direct link between these exposures and their physical health. Instead, they attributed physical health issues to the stress of war.
[35]	Hall et al., 2025	Pre-existing vulnerability due to displacement, trauma, and loss<br>--> Increased Exposure to Climate Hazards (e.g., Water insecurity, agricultural disruption)<br>--> Psychological Distress (e.g., Depression, anxiety, PTSD, hopelessness)<br>--> Inability to Adapt<br>--> Continued Cycle of Hardship	Ongoing intervention trial that provides a framework for how mental health can be both an outcome<br>of these combined stressors and a crucial factor in building resilience and improving adaptive capacity. It directly tests the<br>hypothesis that addressing mental health can serve as a mitigation strategy for climate-related food insecurity in<br>a conflict-affected population.
[36]	Azhar et al., 2023	Vulnerability from civil war and ethnic discrimination<br>--> Rapid-Onset Climate Hazard	- The study shows a clear intersection of war and climate change, with a rapid-onset climate event (Cyclone Mocha) compounding the mental health challenges already present

		--> Combined Trauma & Psychological Distress<br>--> Increased prevalence of anxiety, depression, and PTSD<br>--> Barriers to Humanitarian Aid & Recovery (Political interference, discrimination, stigma, lack of infrastructure)<br>--> Continued Psychosocial Stress	from ongoing civil war and displacement.<br>- It highlights how political factors and government responses, such as delaying aid and discrimination, can worsen mental health outcomes and hinder recovery efforts in a post-disaster setting.<br>- The article argues for the urgent need to integrate mental health and psychosocial support (MHPSS) programs into disaster<br>response, as they are often overlooked in favor of immediate needs like food and shelter.<br>- It emphasizes the importance of community-based approaches, like educating leaders and using volunteers with lived experience, to combat the stigma surrounding mental health and ensure effective care.
[37]	Asad et al., 2013	War + floods --> displacement<br>Inhumane living conditions<br>--> vulnerability and mental health decline<br>--> child trauma & abuse	The paper clearly presents the intersection of climate change and conflict causing displacement, which creates conditions that<br>significantly increase the risk of abuse, trauma, and other negative mental health outcomes for children. The paper also highlights how<br>the combined effects of these two drivers of displacement create a vulnerable population living in an environment that lacks resources<br>and support, leading to serious shortcomings in social, emotional, and cognitive development.
[38]	Eboreime et al., 2025	climate shifts<br>(rising temperatures, reduced rainfall, desertification)<br>--> resource scarcity<br>(water and land)<br>--> intensified conflict between communities<br>--> forced displacement<br>--> psychosocial health impacts	- climate change is a "threat multiplier" for conflict and displacement in West Africa.<br>- The paper explicitly links forced migration and the conditions in IDP camps exposed to climate hazards to severe<br>psychosocial health impacts, including high rates of depression, anxiety, and PTSD.
[39]	Maukera & Blignault, 2015	war + climate hazards<br>--> affected livelihood (homelessness)	The study highlights how a population's vulnerability to rapid- and slow-onset

		--> trauma + mental decline<br>--> long term behavioral changes<br>--> violence + substance abuse + self-harm	climate disasters is heightened by the pre-existing conditions of armed conflict<br>and political unrest. This dual vulnerability leads to a breakdown of a<br>population's resilience, as seen in the lingering fear and disruption of social<br>cohesion, which in turn enables a decline in mental health outcomes, such as<br>depression, substance abuse, and interpersonal violence.
[40]	Zafar et al., 2016	War (displacement, violence, damage) + Floods (infrastructure damage, loss of homes)<br>--> humanitarian crisis<br>--> cumulative trauma<br>--> increased risk of depression<br>--> increased perinatal depression in women	This protocol offers a response to the humanitarian crisis caused by both<br>conflict and devastating flood in Pakistan. The proposed interventions aim<br>o address the mental health of a vulnerable population (mothers), which also<br>directly addresses the health of their offsprings.
[41]	Syed Sherif et al., 2011	Drought + Political violence<br>--> displacement + resource scarcity<br>--> trauma + loss<br>--> compounding mental health impacts	The paper provides an overview of the state of mental healthcare in Somalia, a country<br>affected by both climate hazard (drought) and civil war. With a large number of IDPs<br>and refugees, so little awareness of mental health, and the reliance on<br>traditional/religious healers exacerbate the mental health impacts.
[42]	Schuster et al., 2024	Displacement due to conflict + Climate hazards<br>--> humanitarian crisis in refugee camp<br>--> gendered inequities<br>--> exacerbated mental health issues in women	The paper presents a framework where the initial forced displacement<br>caused by persecution in Burma/Myanmar creates a baseline of vulnerability,<br>and then climate hazards like fires, landslides, and food/water insecurity act<br>as compounding stressors. This combined effect exacerbates existing health<br>inequities and contributes to a humanitarian crisis.
[43]	Sanni et al., 2022	War + Climate hazards<br>--> forced displacement	in this paper climate change is described as a "threat multiplier" that can increase

		--> loss of social capital<br>--> poor mental health	the risk of conflict. Both war and climate change, through events like drought, can<br>force people to leave their homes, which is a traumatic experience. This displacement<br>and the related trauma, along with the loss of social networks, contribute to mental<br>health issues such as stress, sadness, loneliness, and emotional disorders
[44]	Ae-Ngibise et al., 2021	Drought + WAR<br>--> Food insecurity<br>--> poor mental health	The review shows a strong association between food insecurity and mental health outcomes,<br>and highlights that food insecurity is often caused by both climate (drought) and war, also<br>displacement plays a huge role
[45]	McMichael, 2014	climate driven drought + monsoon<br>+ Indian ocean warming<br>+ chronic war+displacement<br>--> "camps are now filled to four times their planned capacity, 50% are children (vulnerable)<br>--> direct risk of mental/emotional impacts + trauma<br><br>Also mentions direct effect of children observing their families stressed<br>about climate change	children are a uniquely vulnerable population to the joint effects of climate change<br>and war. The intersection of a climate hazard (drought) and conflict (civil war)<br>is shown to create a compounded crisis of displacement, which directly leads to<br>trauma and mental and physical health issues.
[46]	Weissbecker, 2009	War/Natural disasters --> Displacement<br>--> disrupted services, chronic stress<br>--> Mental Health Outcomes:<br>(PTSD, depression, anxiety, psychosocial distress)	- This paper explicitly highlights that both disasters (linked to climate hazards) and<br>wars independently and jointly contribute to severe, long-term mental health burdens.<br>- Vulnerable groups are at higher risk of persistent psychological distress.<br>- Mental health is framed as a human right, underscoring the importance of<br>integrating psychosocial support into humanitarian response and recovery.

[47]	Javed, 2016	War --> displacement<br>--> vulnerable conditions<br>--> climate hazards worsen conditions<br>--> loss of livelihood (lack of resources/loss of social capital/<br>scarcity of mental health services)<br>--> trauma	The chapter highlights that Pakistan is a region vulnerable to both natural disasters and war, both of<br>which lead to trauma and forced displacement, causing long-lasting psychosocial problems.<br>These impacts are made worse by the scarcity of mental health services in the country, making it difficult to cope with the<br>"continuous and ongoing war and terrorism".
